# Crystal Structures and Cytotoxicity of *ent*-Kaurane-Type Diterpenoids from Two *Aspilia* Species

**DOI:** 10.3390/molecules23123199

**Published:** 2018-12-04

**Authors:** Souaibou Yaouba, Arto Valkonen, Paolo Coghi, Jiaying Gao, Eric M. Guantai, Solomon Derese, Vincent K. W. Wong, Máté Erdélyi, Abiy Yenesew

**Affiliations:** 1 Department of Chemistry, University of Nairobi, P. O. Box 30197, 00100 Nairobi, Kenya; souaibouyaouba@yahoo.fr (S.Y.); sderese@uonbi.ac.ke (S.D.); 2 Department of Chemistry, University of Jyvaskyla, P.O. Box 35, 40014 Jyvaskyla, Finland; arto.m.valkonen@jyu.fi; 3 State Key Laboratory of Quality Research in Chinese Medicine/Macau Institute for Applied Research in Medicine and Health, Macau University of Science and Technology, Macau 999078, China; coghips@must.edu.mo (P.C.); cubix48@163.com (J.G.); bowaiwong@gmail.com (V.K.W.W.); 4 Department of Pharmacology and Pharmacognosy, School of Pharmacy, University of Nairobi, P. O. Box 19676, 00202 Nairobi, Kenya; eguantai@uonbi.ac.ke; 5 Department of Chemistry–BMC, Uppsala University, Husargatan 3, 75237 Uppsala, Sweden; 6 The Swedish NMR Centre, Medicinaregatan 5, 40530 Gothenburg, Sweden; 7 Department of Chemistry and Molecular Biology, University of Gothenburg, 40530 Gothenburg, Sweden

**Keywords:** Asteraceae, *Aspilia pluriseta*, *Aspilia mossambicensis*, *ent*-kaurane diterpenoid, X-ray crystal structure, cytotoxicity

## Abstract

A phytochemical investigation of the roots of *Aspilia pluriseta* led to the isolation of *ent*-kaurane-type diterpenoids and additional phytochemicals (**1**–**23**). The structures of the isolated compounds were elucidated based on Nuclear Magnetic Resonance (NMR) spectroscopic and mass spectrometric analyses. The absolute configurations of seven of the *ent*-kaurane-type diterpenoids (**3**–**6**, **6b, 7** and **8**) were determined by single crystal X-ray diffraction studies. Eleven of the compounds were also isolated from the roots and the aerial parts of *Aspilia mossambicensis*. The literature NMR assignments for compounds **1** and **5** were revised. In a cytotoxicity assay, 12α-methoxy-*ent*-kaur-9(11),16-dien-19-oic acid (**1**) (IC_50_ = 27.3 ± 1.9 µM) and 9β-hydroxy-15α-angeloyloxy-*ent*-kaur-16-en-19-oic acid (**3**) (IC_50_ = 24.7 ± 2.8 µM) were the most cytotoxic against the hepatocellular carcinoma (Hep-G2) cell line, while 15α-angeloyloxy-16β,17-epoxy-*ent*-kauran-19-oic acid (**5**) (IC_50_ = 30.7 ± 1.7 µM) was the most cytotoxic against adenocarcinomic human alveolar basal epithelial (A549) cells.

## 1. Introduction

The genus *Aspilia* belongs to the family Asteraceae. The majority of plants in this family are herbaceous, while trees and shrubs are rare [1]. Plants belonging to the Asteraceae family are found worldwide, except Antarctica [2]. They are found in cooler montane habitats or temperate areas in tropical regions, and are not common dwellers of hot lowland tropical rain forests [1,2]. The family of Asteraceae is one of the largest plant families and the richest in vascular plants in the world. The family has about 1,600–1,700 genera and 24,000–30,000 species [1,3,4]. Plants from the genus *Aspilia* (Asteraceae) occur widely in South, South-West, and West Kenya, from the coast to Lake Victoria. The genus *Aspilia* exhibits biological activities, including antibacterial and antifungal effects, mainly attributed to the presence of kaurane-type diterpenoids [3,5] and sesquiterpene lactones [5,6].

*Aspilia pluriseta* Schweinf has been used in traditional medicine to treat lacerations, bruises and burns, and it is reputed to aid in the healing of cutaneous lesions [7]. The plant is found in Kenya and is commonly known as ‘Dwarf Aspilia’ [7]. The presence of diterpenoids from aerial parts of *A. pluriseta* has been reported previously [8], and four of these diterpenoids exhibited moderate activity against chloroquine-sensitive (D6) and chloroquine-resistant (W2) strains of *Plasmodium falciparum*. The aqueous extract of the plant was also reported by the same authors to exhibit hypoglycemic properties in alloxanized mice. *A. pluriseta* is locally known in Kenya as *Muuti* (Kikuyu), *Wuti* (Kamba), *Ol-oiyabase* (Maasai), and *Shilambila* (Luhya). Many communities in Kenya, as well as some in the rest of Eastern and Southern Africa, use the plant ethnomedically to treat wounds [7].

*Aspilia mossambicensis* (Oliv.) Wild is a shrub native to central and Eastern tropical Africa. The plant is found in the Democratic Republic of Congo, Ethiopia, Kenya, Malawi, Mozambique, Tanzania, Uganda, Zambia, and Zimbabwe [9]. In Eastern Africa, the plant is well known for the treatment of cystitis, gonorrhea, abdominal pain, intestinal worms, and skin infections [9,10,11,12]. The thiophene derivatives, thiarubrines A and B, have previously been isolated from *Aspilia mossambicensis* [9]. The roots of this plant exhibited antibacterial activity, which was suggested to explain its use by wild chimpanzees [9,11]. Herein, we report the phytochemical investigation and the cytotoxicity study of the constituents of *Aspilia pluriseta* Schweinf and *Aspilia mossambicensis* (Oliv.) Wild (Asteraceae).

## 2. Results and Discussion

**Figure 1:**
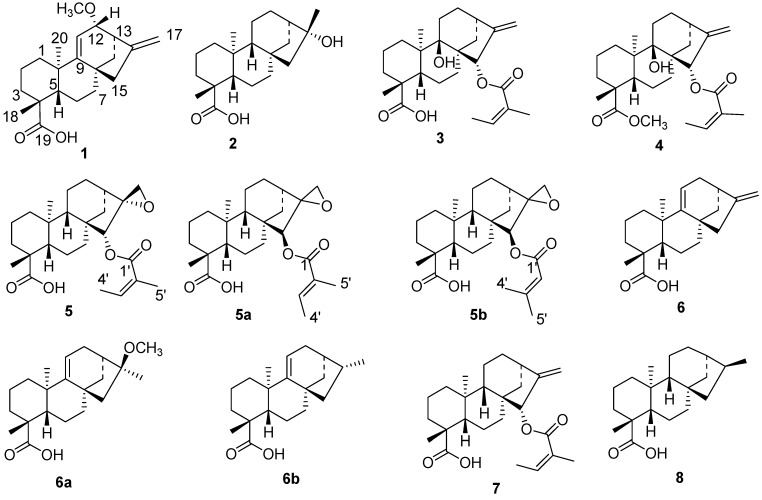
Structures of compounds **1**–**8**.

Compound **1**, [α${]}_{D}^{20}$−88°, was isolated as colorless crystals (m.p. 184–186 °C) from the CH_2_Cl_2_/MeOH (1:1) extract of the roots of *Aspilia pluriseta*. HRMS (Figure S7, Supplementary Material) showed a [M–H]^−^ ion peak at *m/z* = 329.2191, which is in agreement with the molecular formula C_21_H_30_O_3_. The NMR spectra (Table 1) indicated that this compound is a kaurene diterpenoid (Figure 1). The ^1^H–NMR spectrum further revealed the presence of three olefinic protons, namely H-11 (δ_H_ 5.30), H-17a (δ_H_ 4.84) and H-17b (δ_H_ 4.94), suggesting two double bonds. The ^13^C–NMR chemical shifts of C-16 (δ_C_ 152.9) and C-17 (δ_C_ 108.1) are typical of a terminal double bond in an *ent*-kaurene skeleton.

**Figure 2:**
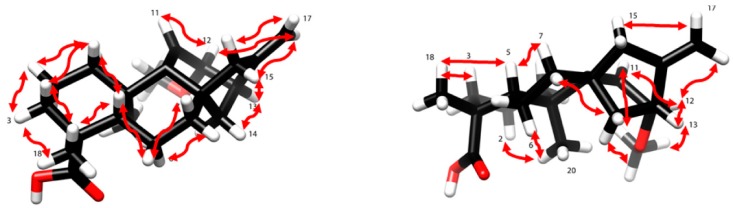
Some of the key NOE correlations observed for compound **1**. The NOESY (Nuclear Overhauser Effect Spectroscopy) spectrum (800 MHz, CDCl_3_, 25 °C, 700 ms mixing time) is shown in the Supplementary Materials.

The second double bond was placed between C-9 (δ_C_ 160.2) and C-11 (δ_H_ 5.30; δ_C_ 115.3) by comparison of the NMR data with that found in the literature [13,14,15]. Signals indicating the presence of a methoxy (δ_H_ 3.34, δ_C_ 56.5) and a carboxylic acid (δ_C_ 183.2) substituent were observed. The Heteronuclear Multiple Bond Correlations (HMBCs) of CH_3_-18 (δ_H_ 1.17), H-3 (δ_H_ 0.93), and H-5 (δ_H_ 1.56) with the carboxy resonance C-19 (δ_C_ 183.2) suggested the location of the carboxy group (C-19) at C-4. Out of the three methyl groups expected in kaurene diterpenoid, only two, i.e., CH_3_-18 (δ_H_ 1.17, δ_C_ 28.2) and CH_3_-20 (δ_H_ 1.01, δ_C_ 23.4), were observed. This corroborated the suggestion of the third methyl group being oxidized to a carboxylic acid (C-19, δ_C_ 183.2). The methoxy group OCH_3_-12 (δ_H_ 3.34) showed HMBC correlation with C-12 (δ_C_ 81.7), whereas H-12 (δ_H_ 3.38) showed HMBC correlation with C-9 (δ_C_ 160.2), C-11 (δ_C_ 115.3), C-13 (δ_C_ 43.7), C-16 (δ_C_ 152.9), and OCH_3_-12 (δ_C_ 56.5). Furthermore, CH_3_-20 (δ_H_ 1.01) showed HMBC correlation with C-1 (δ_C_ 40.6), C-5 (δ_C_ 46.1), the olefinic carbon C-9 (δ_C_ 160.2), and C-10 (δ_C_ 38.9). This confirmed that the second double bond in the molecule is located at C-9. Moreover, the HMBC correlation of CH_2_-14 (δ_H_ 1.31, 1.58) with a deshielded carbon C-12 (δ_C_ 81.7) is in agreement with OCH_3_ being connected to C-12. The above findings confirmed the identity of compound **1** as a C-12-methoxy substituted *ent*-kaur-9(11),16-dienoic acid derivative. The relative configuration at C-12 was deduced from the Nuclear Overhauser Effect (NOE) of OCH_3_-12 (δ_H_ 3.34) to H-13 (δ_H_ 2.89) (Figure 2), indicating them to be syn-oriented, and hence OCH_3_-12 to be α-oriented. It should be noted that H-12 (δ_H_ 3.38) also showed a weak NOE to H-13 (δ_H_ 2.89), which is expected in a strained ring system. The proposed configuration at C-12 is further corroborated by the NOE of H-12 (δ_H_ 3.38) with H-14b (δ_H_ 1.58). The NOE of H-12 (δ_H_ 3.38 ppm) with H-17b (δ_H_ 4.94 ppm) supported H-12 to be β-oriented, and hence OCH_3_-12 to be α-oriented. Based on the above spectroscopic evidence, compound **1**, 12α-methoxy-*ent*-kaur-9(11),16-dien-19-oic acid, (Figure 1) was identified as (4*R*,4a*S*,6a*S*,9*R*,10*S*,11b*R*)-10-methoxy-4,11b-dimethyl-8-methylene-1,2,3,4,4a,5,6,7,8,9,10,11b-dodecahydro-6a,9-methanocyclohepta[*a*]naphthalene-4-carboxylic acid. This compound has previously been reported both as synthetic derivative [13] and as a natural product [14,15]. However, our NMR data assignment differs from that reported in the literature [13] for its C-3 and C-7. The accuracy of the corrected assignment, given in Table 1, is corroborated by the HMBC correlations of H-3 and H-7 (Table 1), by the HSQC crosspeaks of CH_2_-3 (δ_H_ 0.93/2.08) to C-3 (δ_C_ 38.1) along with the TOCSY(Total Correlation Spectroscopy) correlations of CH_2_-3 to CH_2_-1 (δ_H_ 1.14/1.90) and CH_2_-2 (δ_H_ 1.43/1.79), and by the HSQC (Heteronuclear Single Quantum Correlation) crosspeaks of CH_2_-7 (δ_H_ 1.42/1.95) to C-7 (δ_C_ 28.9) along with the TOCSY correlations of CH_2_-7 to CH_2_-6 (δ_H_ 1.82/2.43) and H-5 (δ_H_ 1.56) (Figures S4–S6, Supplementary Material). Besides the compound having been reported earlier, it is unlikely to be an extraction artifact as the extraction (with CH_2_Cl_2_/MeOH, 1:1) has been performed at low temperature at neutral pH that does not promote formation of methyl ethers. The compound has been detected in the crude extract indicating that the compound is a natural product and has not formed during the chromatographic isolation.

**Figure 3:**
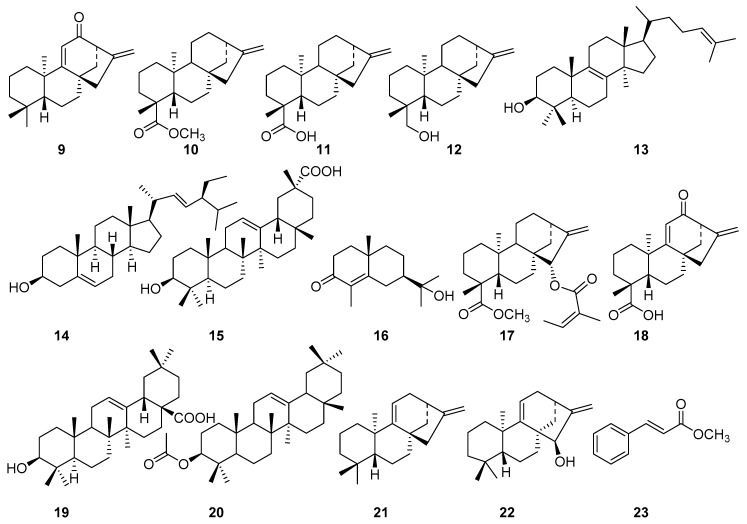
Structures of compounds **9**–**23**.

Additional compounds (Figure 1 and Figure 3) isolated from the roots of *Aspilia pluriseta* include (16*R*)-hydroxy-*ent*-kauran-19-oic acid (**2**) [16,17], 9β-hydroxy-15α-angeloyloxy-*ent*-kaur-16-en-19-oic acid (**3**) [18], methyl-9β-hydroxy-15α-angeloyloxy-*ent*-kaur-16-en-19-oate (**4**) [19], 15α-angeloyloxy-16β,17-epoxy-*ent*-kauran-19-oic acid (**5**) [20], *ent*-kaur-9(11),16-dien-19-oic acid (**6**) [21], 15α-angeloyloxy-*ent*-kaur-16-en-19-oic acid (**7**) [22], *ent*-kaur-9(11),16-dien-12-one (**9**) [23] and methyl-*ent*-kaur-16-en-19-oate (**10**) [24]. The aerial part of *Aspilia pluriseta* contained *ent*-kaur-16-en-19-oic acid (**11**) [21,25], *ent*-kaur-16-en-19-ol (**12**) [26], lanosterol (**13**) [27], stigmasta-5,22(E)-dien-3β-ol (**14**) [28], 3β-hydroxy-olean-12-en-29-oic acid (**15**) [29], and carissone (**16**) [30].

Similar phytochemical investigation of the roots of *Aspilia mossambicensis* resulted in the isolation of methyl-15α-angeloyloxy-*ent*-kaur-16-en-19-oate (**17**) [24,31], 12-oxo-*ent*-kaur-9(11),16-dien-19-oic acid (**18**) [32], (16S)-*ent*-kauran-19-oic acid (**8**) [33], oleanolic acid (**19**) [34] (Figure 3), and compounds **3**–**5**. The aerial part of *A. mossambicensis* afforded compound **6** [21,26], 3β-acetyloxy-olean-12-ene (**20**) [35,36] *ent*-kaur-9(11),16-diene (21) [37], 15*a*-hydroxy-kaur-9(11),16-diene (**22**) [38], and methyl cinnamate (**23**) [39].

**Figure 4:**
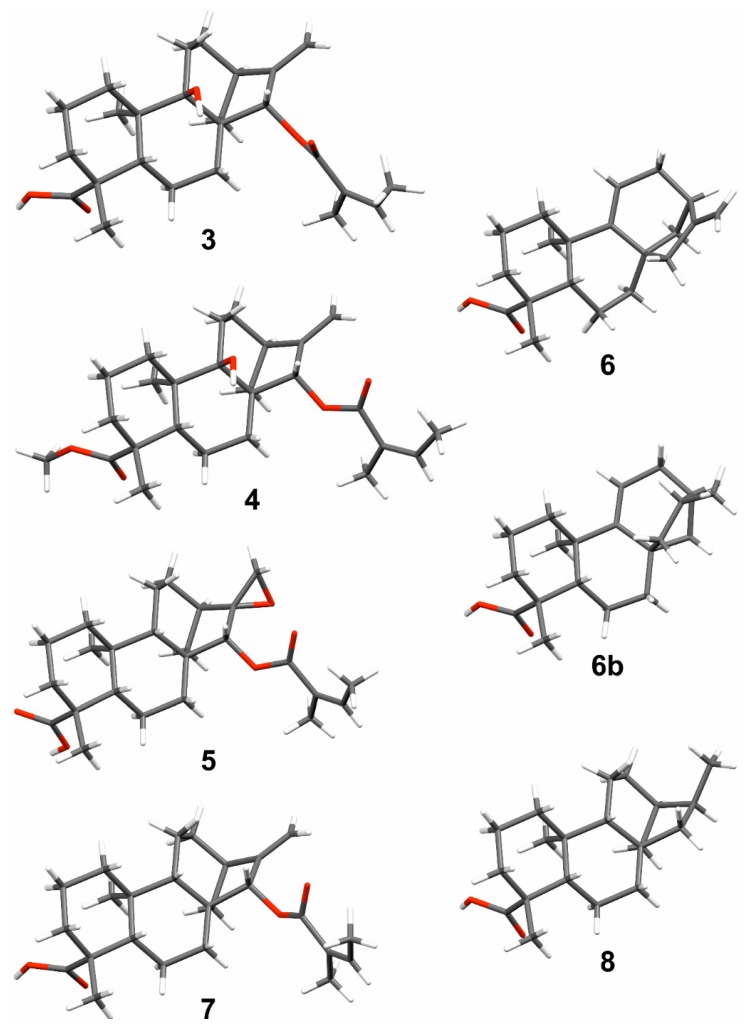
Crystal structure representations of compounds **3**–**8**. The structures were deposited with the following CCDC (Cambridge Crystallographic Data Centre) codes: **3** (1868318), **4** (1868319), **5** (1868321), **6** (1868320), **6b** (1868324), **7** (1868323), and **8** (1868322).

The crystal structures for compounds **3**–**8** (Figure 4) are also reported here, where the identities of the compounds were confirmed, and the absolute configurations established. In the crystal state, compound **3** exhibited a continuous network, involving intermolecular O(9)-H···O(19a) and O(19b)-H···O(21) hydrogen bonds. Similar O(9)-H···O(19a) hydrogen bonding motif was found in compound **4**, which also showed static disorder exhibiting two different spatial orientations of (*Z*)-2-methylbut-2-enoyl group in an approximately 1:1 ratio. Compounds **5**–**8** showed common double hydrogen bonding motifs for carboxylic acids leading to the formation of hydrogen-bonded pairs.

Crystal structure of compound **6** was determined at 120 K, which corresponds to the previously reported structure at room temperature [40]. Crystal structure analysis of a synthetic derivative of compound **6** led to the identification of *ent*-kaur-9(11)-en-19-oic acid (**6b**). In the structure **6b**, there is a slight (~10%) disorder in the main ring system, where carbons C7–C17 have different positions giving a shade of different conformations for the corresponding aliphatic rings. The data quality for compound **7** is slightly deficient and the absolute structure of it could not be justified on the basis of data. The X-ray diffraction data parameters, thermal ellipsoid diagrams, and hydrogen bonding geometries are presented in the Supplementary Information section of this article.

In most of the *ent*-kaurane-type diterpenoids (except for compounds **9**, **21,** and **22**) isolated from the two *Aspilia* species, *A. pluriseta* and *A*. *mossambicensis,* the α-methyl group at C-4 (C-19) is oxidized into carboxylic acid or methyl ester groups, which could be a characteristic feature of the genus *Aspilia* [3,8]. There are also examples where oxidation has occurred at C-12 (compounds **1, 9** and **18**), C-16 (compound **2**), C-9 (compounds **3** and **4**), and C-15 (compounds **4**, **5**, **6,** and **18**). In agreement with the literature [36], oxidation has not been observed at other carbon atoms in *ent*-kaurane-type diterpenoids of *Aspilia* species.

Some *ent*-kaurane-type diterpenoids, including 16,17-epoxy-15β-tigloyloxy-*ent*-kauran-18-oic acid (**5a**) and 16,17-epoxy-15β-senecioyloxy-*ent*-kauran-18-oic acid (**5b**), were reported earlier from *Aspilia pluriseta* [8]. These compounds were reported to have the 15β**-tigloyloxy and 15β-senecioyloxy groups, respectively, occupying the less favorable orientation [8]; however, the authors have not provided evidence for these proposals. Our single crystal X-ray analyses have shown that the C-15 substituent of compounds **3**, **4**, **5,** and **7** is an angeloyloxy group occupying the more favourable-15α-position (Figure 4). In fact, the proposed stereochemical assignment of *ent*-kaurane-type diterpenoids reported from this genus, particularly in highly functionalized compounds, lacks evidence. We have filled this knowledge gap by determining the absolute configuration of seven *ent*-kaurane-type diterpenoids, as shown in Figure 4, using single crystal X-ray analyses.

The ^13^C-NMR data of compound **5** (Table 2) is in close agreement to that previously reported in the literature [20], except for the chemical shift assignment of C-2 and C-12 (Table 2). Related structures, **5a** and **5b**, have been proposed for two compounds earlier reported [8] from *Aspilia pluriseta.* The ^13^C-NMR assignment (Table 2) for these compounds differs from our assignment, which is based on 2D NMR correlations, despite the common 16,17-epoxy-15**-oxy-*ent*-kauran-18-oic acid skeleton (Figure 1). The ^13^C-NMR chemical shifts of C-4’ (δ_C_ 27.4) and C-5’ (δ_C_ 20.8) in compound **5a** do not support a tigloyloxy group at C-15, as proposed in Reference [8]; methyl carbon atoms in such group are expected to resonate at ~14 ppm (for C-4’) and at ~11 ppm (for C-5’), based on chemical shift prediction [41] and previous literature [14]. The NMR spectra of compound **5b** that are given in the supporting information of [8] are of low quality and do not allow confirmation of the proposed assignment. It should be noted that the numbering used in this paper does not follow the literature convention [3]. Hence, the carboxylic group of **5b** and of its structural analogues should not be assigned as C-18, but rather as C-19, following reference [3]. Overall, several details reported [8] for these compounds appear debatable, and consequently so are the proposed structures. To avoid such uncertainties, the NMR assignments of all compounds discussed in this paper are presented in Tables S1 and S2 in the Supplementary Material.

Most of the compounds isolated in this study were assayed against two normal (BEAS-2B and LO_2_) and two cancer (A549 and Hep-G2, Table 3) cell lines. Of the fifteen compounds tested, **1**, **3**, **5**, **9,** and **18** showed cytotoxicity towards some of the cell lines. Compound **1** showed selective activity against the human hepatoma (HepG2) cancer cell line without significant toxicity to other cell lines (IC_50_ above 100 μM). Compound **3** was moderately cytotoxic against the Hep-G2 cancer cell line (IC_50_ = 24.7 ± 2.8), but also showed weak cytotoxicity towards the normal cell lines LO_2_ (IC_50_ = 57.2 ± 1.2) and BEAS-2B (IC_50_ = 89.9 ± 2.0 µM), indicating low selectivity. Compound **5** (IC_50_ = 30.7 ± 1.7 µM) was the most active against the A549 cell line, followed by compound **18** (IC_50_ = 80.5 ± 1.8 µM). The other compounds tested, i.e., **2**, **4**, **6**, **7, 10**, **11**, **14,** or **17**, did not exhibit significant cytotoxicity (IC_50_ above 100 μM). The lack of cytotoxicity against the two normal cell lines of these diterpenoids is valuable information, considering that some kaurane-type diterpenoids from *Aspilia* species have showed antibacterial and antifungal effects [5].

## 3. Materials and Methods

### 3.1. General Experimental Procedures

NMR spectra were acquired on a Bruker Avance II 600 MHz, a Bruker Avance III HD 800 MHz (Bruker BioSpin AG, Fällanden, Switzerland) or a Varian Unity 500 MHz (Varian Inc, Palo Alto, CA, USA) NMR spectrometer, using the residual solvent peaks as a reference. The spectra were processed using the software MestReNova (version 10.0, Mestrelab Research S.L., Santiago de Compostela, Spain) Coupling constants (*J*) are given in Hz. EI-MS and LC-MS were carried out using 70 eV ionization electron voltage on a Micromass GC-TOF spectrometer (Micromass, Wythenshawe, Waters Inc., UK). TLC (Thin Layer Chromatography) was carried out on Merck pre-coated silica gel 60 F_254_ plates (Merck, Darmstadt, Germany). Preparative TLC was performed on 20 × 20 cm glass plates, pre-coated with silica gel 60F_254_ with thicknesses of 0.25 to 1 mm. Column chromatography was run on silica gel 60 Å (70–230 mesh). Gel filtration was performed on Sephadex LH-20(Merck, Darmstadt, Germany).

### 3.2. X-ray Diffraction Analyses

The single crystal X-ray diffraction data were collected using Agilent Super-Nova (Agilent Technologies, Wrocław, Poland) dual wavelength diffractometer with a micro-focus X-ray source and multilayer optics monochromatized Cu-Kα (λ = 1.54184 Å) radiation. Program *CrysAlisPro* [42] was used for the data collection and reduction. The intensities were corrected for absorption using analytical face index absorption correction method. The structures were solved with intrinsic phasing method (*SHELXT* [43]) and refined by full-matrix least squares on *F*^2^ with *SHELXL-2018/3* [44]. Anisotropic displacement parameters were assigned to non-H atoms. All C-H hydrogen atoms were refined using riding models. Hydroxy hydrogens were found from electron density maps and restrained to the proper distance from oxygen atom (0.84 Å). All hydrogen atoms were refined with *U_eq_*(H) of 1.5 × *U_eq_*(C,O) for hydroxy and terminal methyl groups or 1.2 × *U_eq_*(C) for other C-H groups. Further geometric least-squares restraints (s = 0.02) were applied to structures **4**, **6b,** and **7** to obtain more chemically reasonable bond distances between disordered atoms. Anisotropic displacement parameters of few disordered or terminal atoms were restrained (s = 0.01, st = 0.02) to be more equal in structures **3**, **4**, **6b,** and **7**. CCDC 1868318-1868324 contains the supplementary crystallographic data for this paper. These data can be obtained free of charge via http://www.ccdc.cam.ac.uk/conts/retrieving.html (or from the CCDC, 12 Union Road, Cambridge CB2 1EZ, UK; Fax: +44 1223 336033; E-mail: deposit@ccdc.cam.ac.uk). 

### 3.3. Plant Materials

The roots and aerial parts of *Aspilia pluriseta* and *Aspilia mossambicensis* (Asteraceae) were collected from Ngon’g Forest, Kenya. The plants were identified by Mr. Patrick B. Chalo Mutiso, of the Herbarium, School of Biological Sciences, University of Nairobi, Kenya, where voucher specimens (SY2015/04 for *Aspilia pluriseta,* and SY2015/05 for *Aspilia mossambicensis*) were deposited.

### 3.4. Extraction, Isolation and Derivatization

The air-dried and ground roots of *A. pluriseta* (0.8 kg) were extracted with CH_2_Cl_2_/MeOH (1:1) for 24 h affording 47 g of extract. A portion of the extract (35 g) was subjected to column chromatography on silica gel (700 g) and eluted with *hexane* containing increasing amounts of EtOAc. The fraction eluted with 10% EtOAc in *hexane* was purified by crystallization from acetone affording compound **1** (36 mg) [9]. Crystallization (from acetone) of the combined fractions eluted with 5–10% EtOAc in *hexane* afforded compound **2** (47 mg) [12,13]. Preparative TLC separation of the fraction eluted with 15% EtOAc in *hexane* yielded 22 mg of compound **3** [14] and 31 mg of compound **4** [15]. Compound **5** (97 mg) [16] was obtained from the fraction eluted with 30% EtOAc in *hexane*, after purification over Sephadex LH-20 (CH_2_Cl_2_/MeOH; 1:1). Fractional crystallization (from acetone) of the combined fractions eluted with 35–40% EtOAc in *hexane* afforded 218 mg of compound **6** [17] and 89 mg of compound **7** [18]. Purification of the fraction obtained with 60% EtOAc in *hexane* on Sephadex LH-20 (eluted with CH_2_Cl_2_/MeOH; 1:1) led to the isolation of *ent*-kaur-9(11),16-dien-12-one (**9**, 27 mg) [19] and methyl-*ent*-kaur-16-en-19-oate (**10**, 36 mg) [20]. The isolated compounds have been observed in the crude extract, ahead of chromatographic separation, by TLC. This confirmed their presence in the crude extracts, and thus, these are natural products and were not formed during the chromatographic separation.

The air-dried and ground aerial part of *A. pluriseta* (0.8 kg) was extracted, as described above, giving 53 g of crude extract. A portion of the extract (40 g) was subjected to column chromatography on silica gel (800 g) and eluted with *hexane* containing increasing amounts of EtOAc. The fractions eluted with 5–15% EtOAc in *hexane* were combined and purified on Sephadex LH-20 (eluted with CH_2_Cl_2_/MeOH; 1:1) affording lanosterol (**13**, 58 mg) [27] and stigmasta-5,22(E)-dien-3β-ol (**14**, 71 mg) [28]. Elution of the main silica gel column with 20% EtOAc in *hexane* afforded 42 mg of 3β-hydroxy-olean-12-en-29-oic acid (**15**) [29]. The fraction eluted with 30% EtOAc in *hexane* led to the isolation of *ent*-kaur-16-en-19-oic acid (**11**, 367 mg) [17,21] and *ent*-kaur-16-en-19-ol (**12**, 32 mg) [8,26]. Purification of the fraction eluted with 40% EtOAc in *hexane* over Sephadex LH-20 (CH_2_Cl_2_/MeOH; 1:1) yielded carissone (**16**, 26 mg) [30].

The air-dried and ground roots (0.7 kg) of *Aspilia mossambicensis* were extracted with CH_2_Cl_2_/MeOH (1:1) by cold percolation (4 × 24 h) to give gummy brown extract (47 g). A portion of the extract (40 g) was subjected to column chromatography on silica gel (800 g) and eluted with *hexane* containing increasing amounts of EtOAc. Fractional crystallization of the eluent with 25% EtOAc in *hexane* led to the isolation of 64 mg of methyl-15α-angeloyloxy-*ent*-kaur-16-en-19-oate (**17**) [24,31] and 23 mg of 12-oxo-*ent*-kaur-9(11), and 16-dien-19-oic acid (**18**) [32]. Purification of the mother liquor on Sephadex LH-20 resulted in the separation of (16*S*)-*ent*-kauran-19-oic acid (**8**, 17 mg) 33] and oleanolic acid (**19**, 28 mg) [34]. Elution of the column with 40% EtOAc in *hexane* led to the isolation of additional amounts of compounds **3** (14 mg), **4** (7 mg), and **5** (10 mg).

The aerial part (1.0 kg) of *A. mossambicensis* was extracted as above, yielding 82 g of crude extract. A portion of this extract (40 g) was subjected to column chromatography over silica gel (800 g) and eluted with *hexane* containing increasing amounts of EtOAc. Elution of the column with 20% EtOAc in *hexane* led to the isolation of 24 mg of 3β-acetyloxy-olean-12-ene acetate (**20**) [35,36]; elution with 25% EtOAc in *hexane* afforded 73 mg of *ent*-kaur-9(11),16-diene (**21**) [39]. Elution with 30–40% EtOAc in *hexane* resulted in a mixture of two compounds. These were separated by column chromatography on Sephadex LH-20 (eluted with CH_2_Cl_2_/MeOH; 1:1), yielding 21 mg of 15α-hydroxy-kaur-9(11),16-diene (**22**) [38], and 15 mg of methyl cinnamate (**23**) [40].

Derivatization of compound **6**. A concentrated H_2_SO_4_ (2 drops) was added to MeOH (2 mL) solution of compound **6** (0.05 g, 0.165 mmol). The reaction mixture was stirred at 25 °C for 18 h, after which the mixture was poured onto water (20 mL) and extracted with ethyl acetate (3 × 20 mL). The combined organic layer was dried over MgSO_4_, and was then filtered and concentrated in a rotary evaporator, giving 37 mg of **6a** [45].

To hydrogenate *ent*-kaur-9(11),16-dien-19-oic acid (**6**), a solution was obtained by dissolving 100 mg in 20 mL ethanol, in a 50 mL round-bottomed flask, and 5% palladium on charcoal (30 mg) was then added to the solution. The flask was sealed with an airtight rubber stopper. Nitrogen gas was bubbled through the solution to eliminate traces of air using a small syringe inserted through the rubber stopper. Hydrogen gas was then bubbled into the system for 2 days at room temperature. The reaction mixture was filtered and the solvent evaporated yielding compound **6b** (87 mg).

*12α-Methoxy-ent-kaur-9(11),16-dien-19-oic Acid* (**1**) colorless crystals, m.p. 158%–160 °C. [α${]}_{D}^{20}$ −88 (*c* 0.25, acetone), ^1^H and ^13^C-NMR (CD_2_Cl_2_) data (Table 1). ESIMS, *m*/*z* (rel. int.) 329 (12, [M–H]^−^), 315 (11), 299 (100), 253 (71), 281 (10), 171 (18), 182 (3). HRMS [M–H]^−^ *m/z* 329.2191 C_21_H_29_O_3_ (Calculated: 329.2117).

*15α-Angeloyloxy-ent-kaur-16α,17-epoxy-ent-kauran-19-oic Acid* (**5**). Colorless crystals, m.p. 242–243 °C. ^1^H and ^13^C-NMR (CDCl_3_) data, see Table 2. ESIMS, *m/z* (rel. int): 417 (100, [M+H]^+^), 317 (80), 299 (71), 271 (64), 253 (23).

### 3.5. Cell Culture

Reagent and cells. Adenocarcinomic human alveolar basal epithelial (A549) and human hepatoma (HepG2) cancer cell lines, immortalized normal human liver (LO_2_), human bronchial epithelial (BEAS-2B), and fibroblast-like CCD19Lu cells were purchased from ATCC (ATCC, Manassas, VA, USA). The cells were cultured in RPMI-1640 medium supplemented with 10% fetal bovine serum and antibiotics: Penicillin (50 U/Ml) and streptomycin (50 μg/mL; Invitrogen, Paisley, Scotland, UK). All cells were incubated at 37 °C in a 5% humidified CO_2_ incubator). All test compounds were dissolved in DMSO at a final concentration of 50 mM and stored at −20 °C before use.

### 3.6. Cytotoxicity Assay

Cytotoxicity was assessed using the 3-[4,5-dimethylthiazole-2-yl]-2,5-diphenyltetrazolium bromide (MTT) (5 mg/mL) assay, as described previously [46]. Briefly, 5 × 10^3^ cells per well were seeded in 96-well plates before drug treatments. After overnight cell culture, the cells were exposed to different concentrations of selected compounds (0.19–100 μM) for 72 h. Cells without drug treatment were used as controls. Subsequently, 10 μL of 5 mg/mL MTT solution was added to each well and incubated at 37 °C for 4 h, followed by the addition of 100 μL solubilization buffer (12 mM HCl in a solution of 10% SDS) and overnight incubation. The absorbance, A_570_ nm, was then determined in each well on the next day. The percentage cell viability was calculated using the expression: % Viability = A_treated_/ A_control_ × 100, and was given as cytotoxicity in Table 3.

## 4. Conclusions

Twenty-three compounds, mostly *ent*-kaurane-type diterpenoids, were isolated from *Aspilia pluriseta* and *A. mossambicensis*. Besides giving a full NMR assignment, the absolute configuration of seven of the isolated compounds was established by single crystal X-ray diffraction analyses. The isolated compounds were tested for their cytotoxicity against four cell lines. Compounds **1**, **3**, **5**, **9**, and **18** showed moderate to weak cytotoxicity against the cell lines. Compound **3** was the most cytotoxic (IC_50_=24.7 ± 2.8 µM) against the human hepatoma (Hep-G2) cancer cell line without toxicity against the tested normal cell lines.

## Figures and Tables

**Table 1:** The ^1^H (800 MHz) and ^13^C-NMR (200 MHz) data for compound **1** acquired in CDCl_3_.

Position	δ_C_ Lit. [13] *	δ_C_	*δ*_H_, mult. (*J* in Hz)	HMBC (^2^*J*, ^3^*J*)
1	38.17	40.6	1.14 *ddd* (13.5, 9.5, 4.2)	C-2, C-3, C-10, C-20
			1.90 *ddd* (13.5, 3.5, 1.4)	C-2, C-3, C-10, C-20
2	18.35	20.0	1.43 *dddd* (14.2, 9.5, 3.9, 3.5)	C-1, C-3, C-4, C-5, C-10
			1.79 *ddddd* (14.2, 11.1, 4.2, 3.5, 1.4)	C-1, C-4, C-5
3	29.03	38.1	0.93 *ddd* (13.4, 11.1, 3.9)	C-1, C-2, C-4, C-18, C-19
			2.08 *ddd* (13.4, 3.5, 3.5)	C-1, C-4, C-5, C-7
4	43.43	44.6		
5	43.81	46.1	1.56 *dd* (11.1, 8.5)	C-4, C-7, C-9, C-10, C-18, C-19, C-20
6	20.07	18.3	1.82 *dddd* (14.2, 10.0, 8.5, 2.5)	C-3, C-4, C-5, C-7, C-10
			2.43 *dddd* (14.2, 11.1, 9.5, 3.5)	C-4, C-5, C-8
7	40.60	28.9	1.42 *ddd* (13.8, 3.5, 2.5)	C-5, C-6, C-8, C-9, C-15
			1.95 *ddd* (13.8, 10.0, 9.5)	C-6, C-8, C-9, C-14, C-15
8	44.66	43.4		
9	160.28	160.2		
10	38.94	38.9		
11	115.42	115.3	5.30 *dd* (4.3, 1.4)	C-8, C-9, C-10, C-12, C-13, C-15, C-20
12	81.79	81.7	3.38 *dd* (4.3, 2.9)	C-9, C-11, C-13, C-16, C-20, OCH_3_-12
13	46.17	43.7	2.89 *dd* (2.9, 1.4)	C-10, C-11, C-12, C-15, C-16
14	40.60	40.5	1.31 *dd* (10.8, 4.3)	C-7, C-8, C-9, C-12, C-13, C-15
			1.58 *dd* (10.8, 2.5)	C-9, C-12, C-13, C-15, C-16
15	47.17	47.1	2.08 *dd* (15.4, 4.3)	C-7, C-8, C-9, C-16, C-17
			2.35 *dd* (15.4, 2.5)	C-7, C-9, C-13, C-14, C-16, C-17
16	153.00	152.9		
17	108.12	108.1	4.84 *dd* (3.0, 1.6)	C-12, C-13, C-15, C-16
			4.94 *dd* (3.0, 1.6)	C-12, C-13, C-15, C-16
18	28.22	28.2	1.17 *s*	C-3, C-4, C-5, C-8, C-19
19	182.98	183.2		
20	23.41	23.4	1.01 *s*	C-1, C-5, C-9, C-10
OCH_3_-12	56.53	56.5	3.34 *s*	C-12

* CDCl_3_ at 100 MHz [13].

**Table 2:** The literature reported NMR data for **5**, **5a,** and **5b** and the ^1^H (800 MHz) and ^13^C-NMR (200 MHz) data for compound **5** acquired in CDCl_3_.

Position	5 [20]	5a [8]	5b [8]	5	5
	δ_C_	δ_C_	δ_C_	δ_C_	*δ*_H_, mult. (*J* in Hz)
1	41.2	40.6	40.6	40.6	0.80 *ddd* (7.2, 7.1, 1.3)
					1.86* *dd* (2.9, 1.4)
2	28.9	19.8	19.0	19.7	1.55 *ddd* (7.3, 3.6, 2.4)
					1.75 *dd* (3.7, 3.6)
3	37.7	36.7	36.4	37.6	0.96 *ddd* (13.7, 13.6, 4.3)
					2.11 *dd* (13.7, 3.1)
4	43.6	46.9	47.8	43.5	
5	56.7	20.3	56.6	56.5	1.16 *dd* (9.1, 7.1)
6	19.0	41.2	20.3	20.8	1.76 *ddd* (5.7, 3.4, 2.1)
					1.86* *ddd* (3.4, 3.4, 2.7)
7	35.4	47.8	41.2	35.3	1.25 *ddd* (14.4, 13.9, 4.4)
					1.79 *ddd* (13.8, 13.2, 4.3)
8	47.9	52.9	43.6	47.8	
9	52.9	43.6	53.0	52.8	1.28 *dd* (13.8, 3.8)
10	39.8	56.6	39.8	39.7	
11	19.8	20.8	19.8	18.9	1.40 *ddd* (13.8, 3.4, 3.4, 3.1)
					1.81 *dd* (13.8, 4.3)
12	20.8	28.9	28.9	28.8	1.50 *ddd* (13.5, 7.8, 7.2)
13	41.2	36.4	35.1	41.1	1.82 *dd* (13.8, 4.4)
14	36.5	37.7	37.7	36.4	1.68 *dd* (14.5, 3.3)
					1.97 *dd* (13.1, 3.4)
15	81.9	81.2	81.2	81.9	4.73 *br s*
16	66.3	66.4	66.4	66.3	
17	49.6	49.6	49.6	49.6	2.78 *dd* (5.6, 1.3)
					3.09 *dd* (5.8, 1.3)
18	28.8	28.9	28.9	28.7	1.28 *s*
19	182.3	182.6	182.6	182.7	
20	15.7	15.8	16.0	15.9	1.03 *s*
1’	167.9	166.5	166.5	167.8	
2’	128.1	129.0	115.9	128.0	
3’	137.3	137.1	156.8	137.3	5.96 *q* (7.1)
4’	15.9	27.4	20.8	15.7	1.96 *d* (1.9)
5’	20.6	20.8	27.4	20.6	*s*

**Table 3:** Cytotoxicity (IC_50_, μM) of compounds isolated from *Aspilia* species against various cell lines.

Compound	Normal Cell Lines		Cancer Cell Lines	
	BEAS-2B	LO_2_	A549	Hep-G2
**1**	>100	>100	>100	27.3 ± 1.9
**2**	>100	>100	>100	>100
**3**	89.9 ± 2.0	57.2 ± 1.2	>100	24.7 ± 2.8
**4**	>100	>100	>100	>100
**5**	>100	>100	30.7 ± 1.7	>100
**6**	>100	>100	>100	>100
**6a**	>100	>100	>100	>100
**6b**	>100	>100	>100	>100
**7**	>100	>100	>100	>100
**9**	>100	75.3 ± 2.8	>100	>100
**10**	>100	>100	>100	>100
**11**	>100	>100	>100	>100
**14**	>100	>100	>100	>100
**17**	>100	>100	>100	>100
**18**	38.6 ± 2.5	30.0 ± 1.7	80.5 ± 1.8	81.3 ± 0.3
Paclitaxel	<0.1	<0.1	0.0033	0.19

## Supplementary Materials

The following are available online: NMR and MS spectra for the new compound **1**, spectral data for the known compounds, single crystal X-ray diffraction data and refinement parameters and hydrogen bonding geometries. The original FIDs and spectra (mnova) of compounds **1**–**23**. The NMReDATA file of **1** [47].
