# Supplementary material for: Crystal Structures and Cytotoxicity of ent-Kaurane-Type Diterpenoids from Two Aspilia Species
Source: Molecules. 2018 Dec 4;23(12):3199. doi: 10.3390/molecules23123199 (PMC6321058; doi:10.3390/molecules23123199)
Supplement: Supplementary file 1 [file molecules-23-03199-s001.pdf]

SUPPLEMENTARY MATERIAL

Crystal Structures and Cytotoxicity of *ent*-Kaurane-Type Diterpenoids from two *Aspilia species*

Souaibou Yaouba, Arto Valkonen, Paolo Coghi, Jiaying Gao, Eric M. Guantai, Solomon Derese, Vincent K. W. Wong, Máté Erdélyi* and Abiy Yenesew*

Contents

1.1. NMR and MS spectra of 12α-methoxy-*ent*-kaur-9(11),16-dien-19-oic acid (1) 2

1.2. NMR and MS spectra of 15α-angeloyloxy-16β,17-epoxy-*ent*-kauran-19-oic acid (5). 5

1.3. Spectroscopic data of the known compounds 2-23. 9

1.4. X-ray diffraction analyses 18

1.5. References 29

The original FIDs for all 2D spectra for compounds **1-23** are available from the corresponding authors.

1.1. NMR and MS spectra of 12α-methoxy-*ent*-kaur-9(11),16-dien-19-oic acid (1)

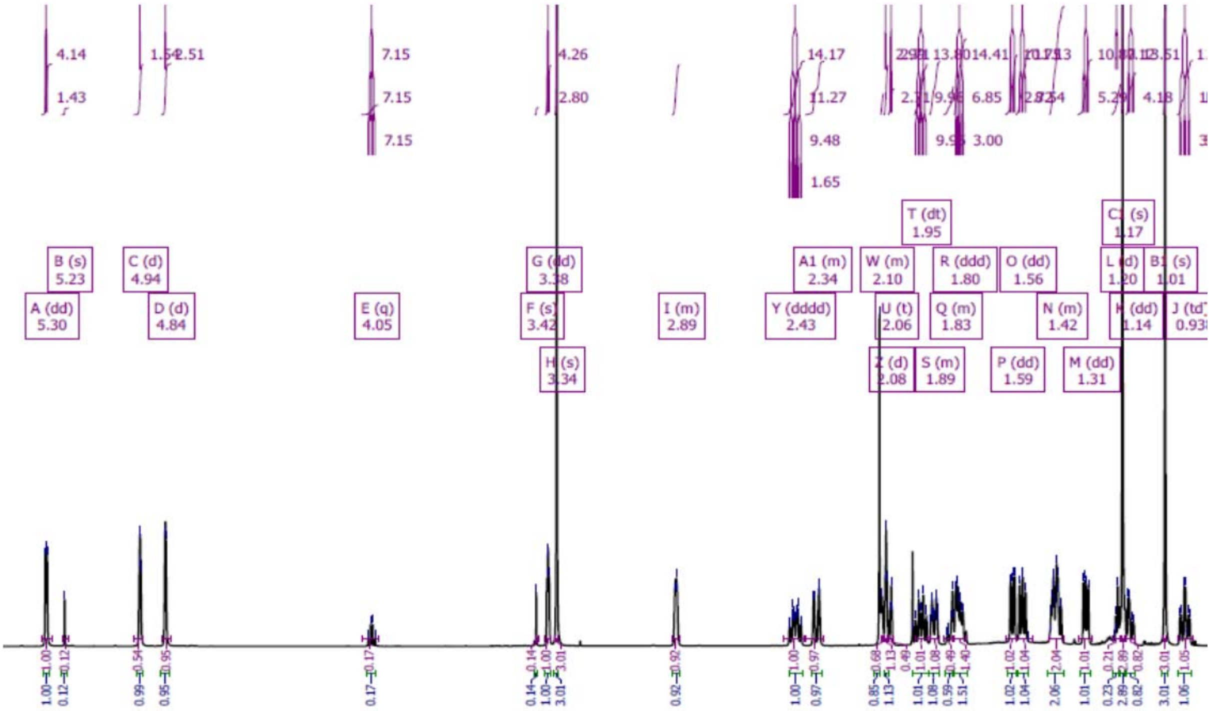

Figure S1. The ^{1}H NMR spectrum of 12α-methoxy-*ent*-kaur-9(11),16-dien-19-oic acid (**1**) observed at 800 MHz for $CDCl_3$ solution at 25 °C. Assignment is given in Table 1.

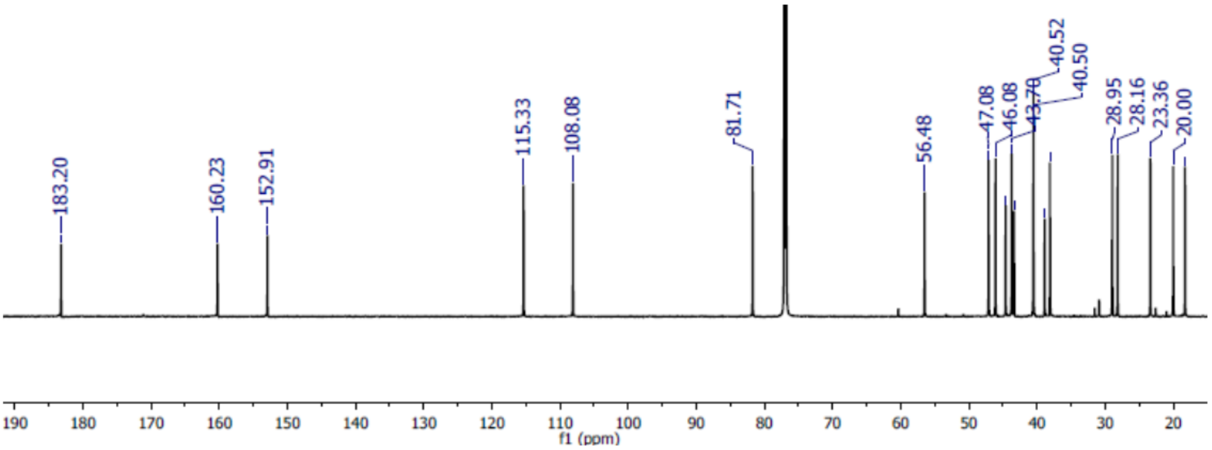

Figure S2. The ^{13}C NMR spectrum of 12α-methoxy-*ent*-kaur-9(11),16-dien-19-oic acid (**1**) observed at 200 MHz for $CDCl_3$ solution at 25 °C. Assignment is given in Table 1.

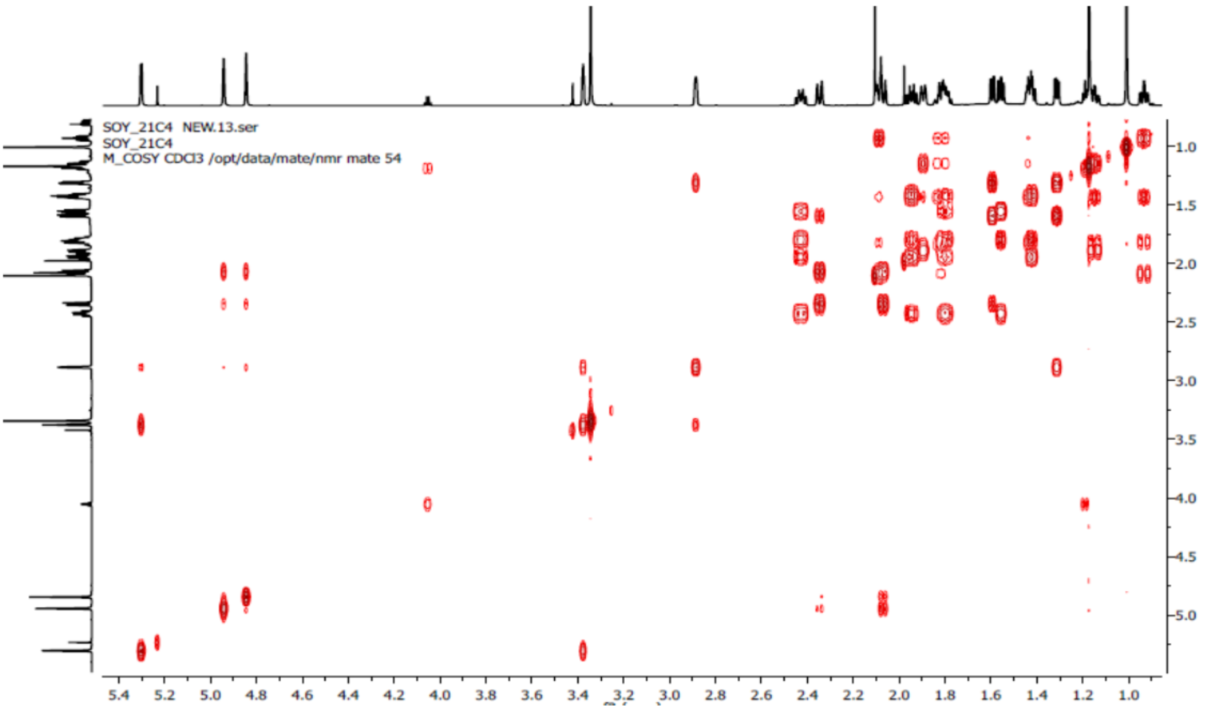

Figure S3. The ^{1}H-^{1}H COSY spectrum of 12α-methoxy-*ent*-kaur-9(11),16-dien-19-oic acid (**1**) observed at 800 MHz for $CDCl_3$ solution at 25 °C.

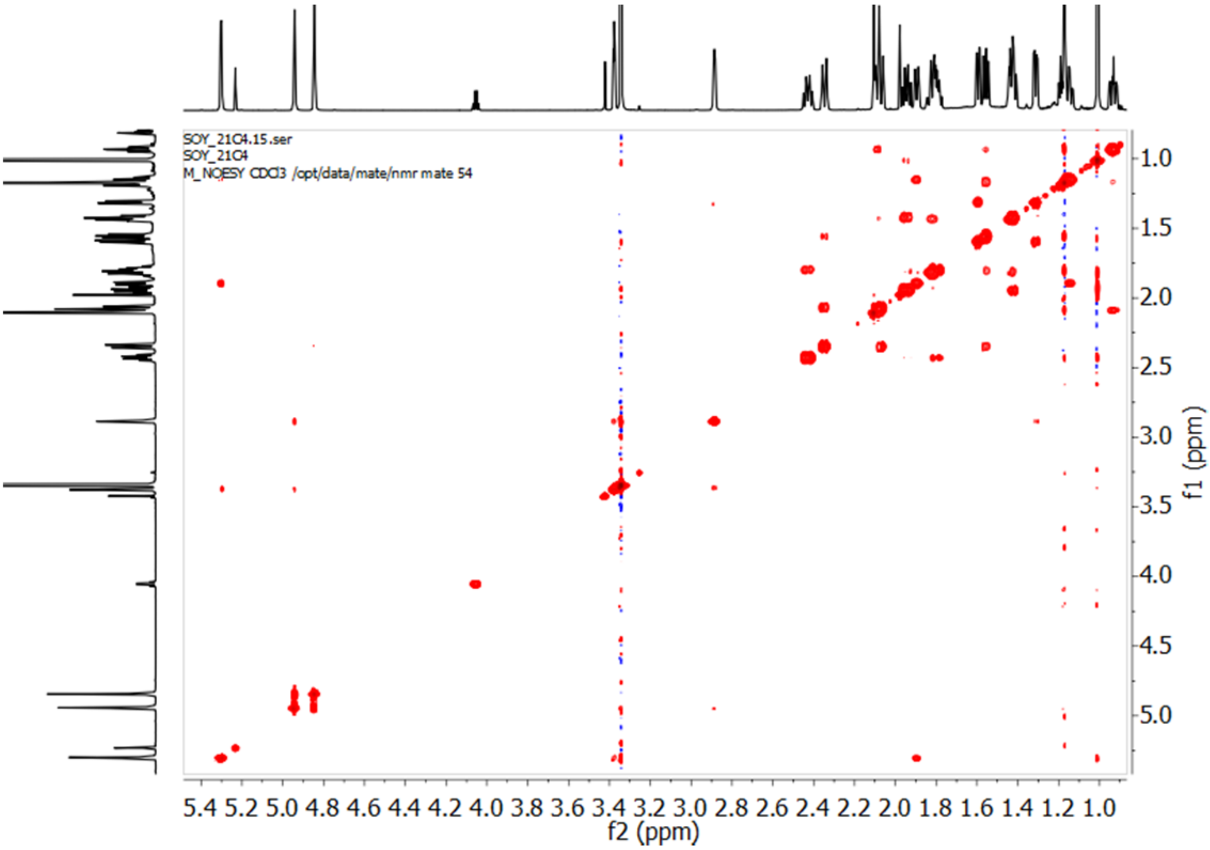

Figure S4. The ^{1}H-^{1}H NOESY NMR spectrum of 12α-methoxy-*ent*-kaur-9(11),16-dien-19-oic acid (**1**) observed at 800 MHz for $CDCl_3$ solution at 25 °C.

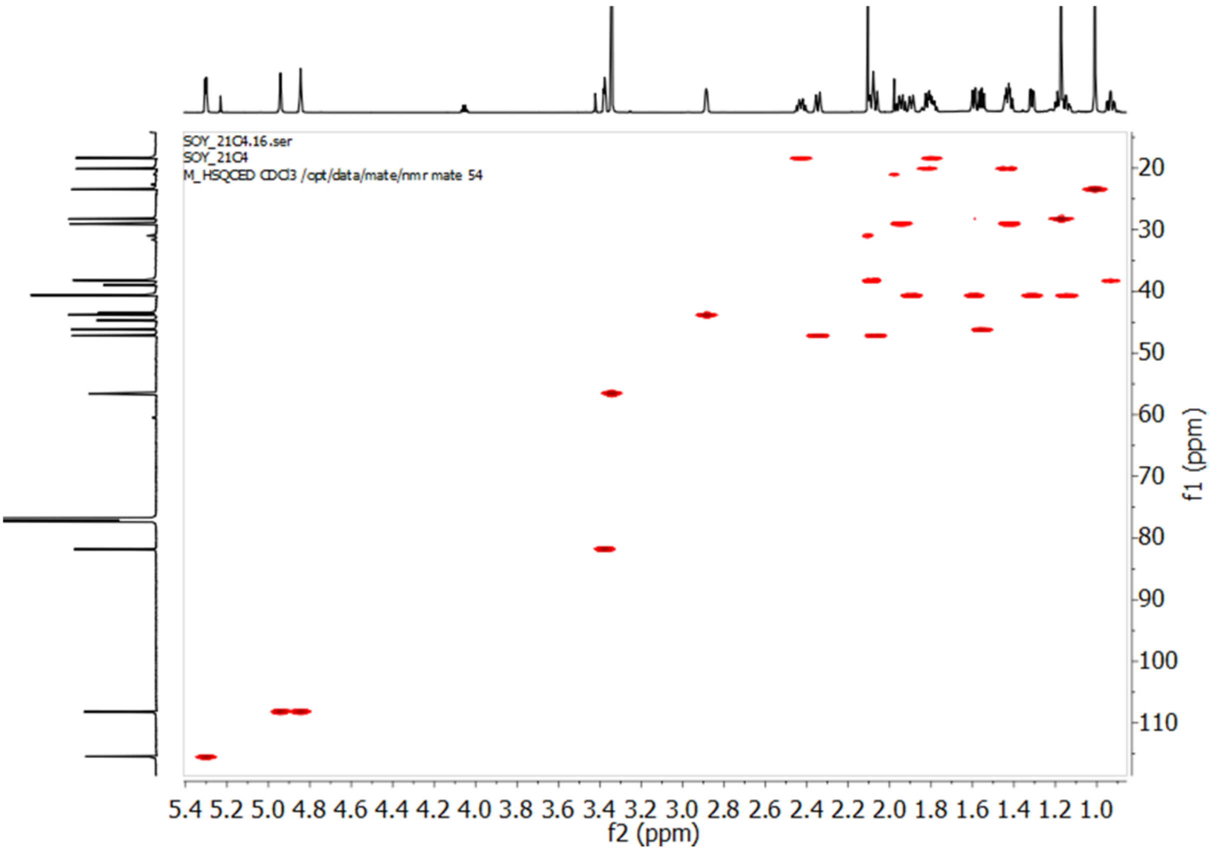

Figure S5. The ^{1}H-^{13}C HSQC NMR spectrum of 12*α*-methoxy-*ent*-kaur-9(11),16-dien-19-oic acid (**1**) observed at 800 and 200 MHz for $CDCl_3$ solution at 25 °C.

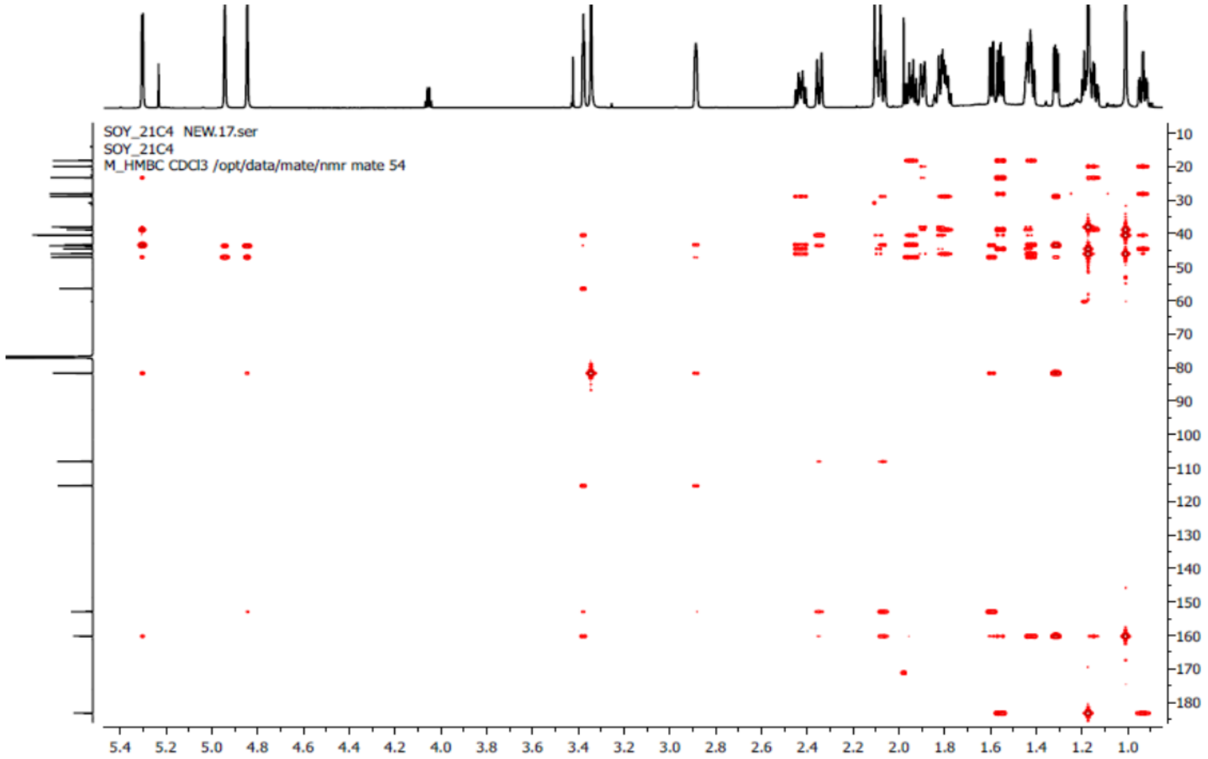

Figure S6. The ^{1}H-^{13}C HMBC NMR spectrum of 12*α*-methoxy-*ent*-kaur-9(11),16-dien-19-oic acid (**1**) observed at 800 MHz for $CDCl_3$ solution at 25 °C.

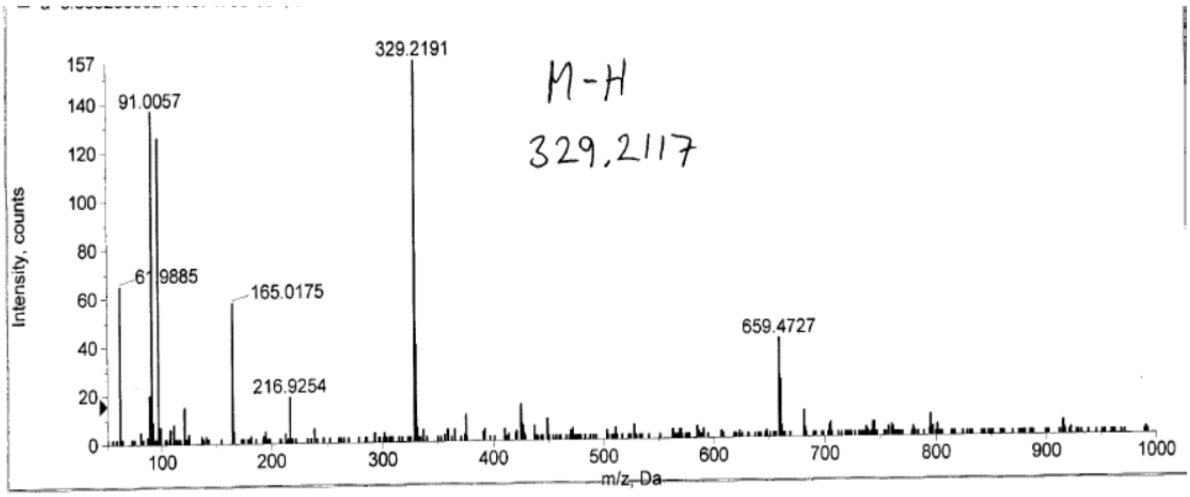

Figure S7. The HRMS (ESI) spectrum of 12α-methoxy-*ent*-kaur-9(11),16-dien-19-oic acid (**1**).

1.2. NMR and MS spectra of 15α-angeloyloxy-16β,17-epoxy-*ent*-kauran-19-oic acid (5).

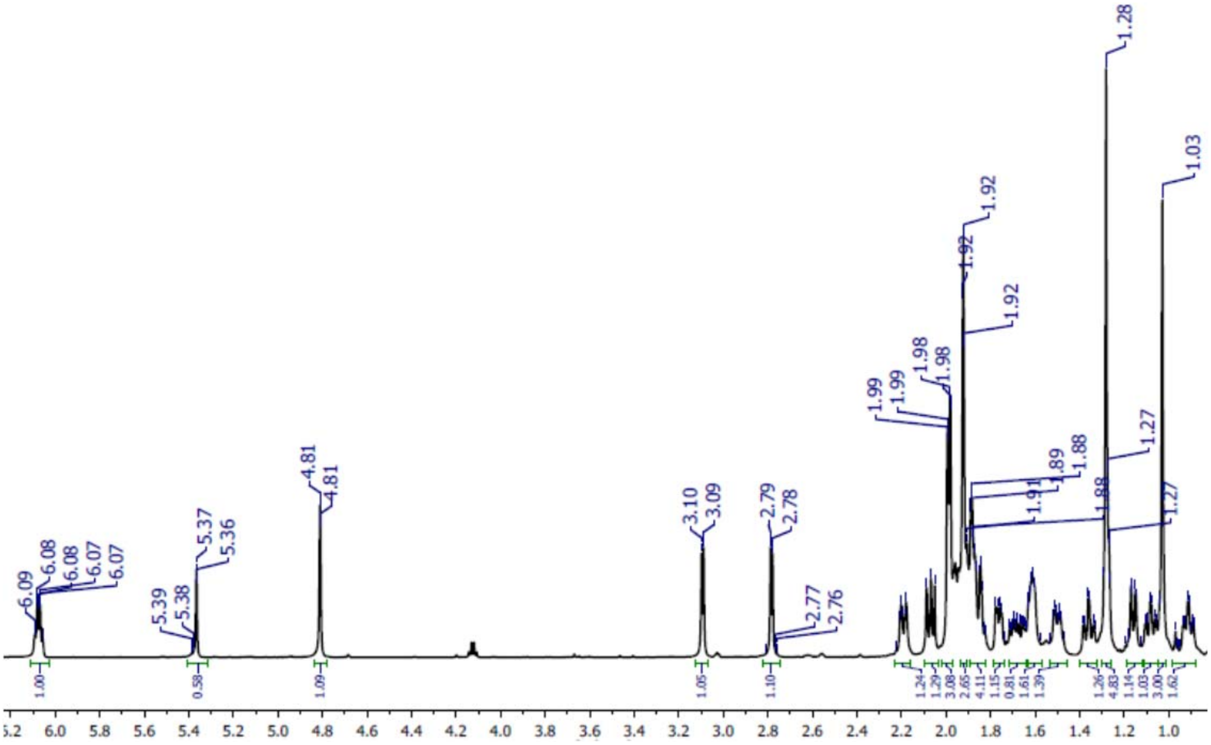

Figure S8.The ^{1}H NMR spectrum of 15α-angeloyloxy-16β,17-epoxy-*ent*-kauran-19-oic acid (**5**) observed at 800 MHz for $CDCl_3$ solution at 25 °C. Assignment is given in Table 1.

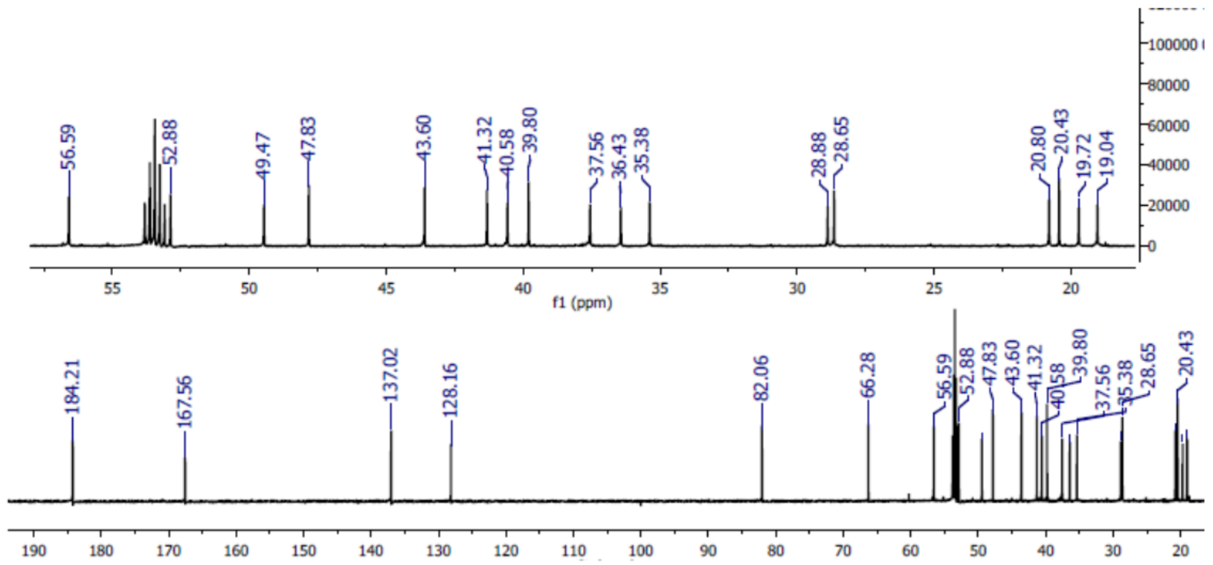

Figure S9.The ^{13}C NMR spectrum of 15α-angeloyloxy-16β,17-epoxy-*ent*-kauran-19-oic acid (**5**) observed at 200 MHz for $CDCl_3$ solution at 25 °C. Assignment is given in Table 1.

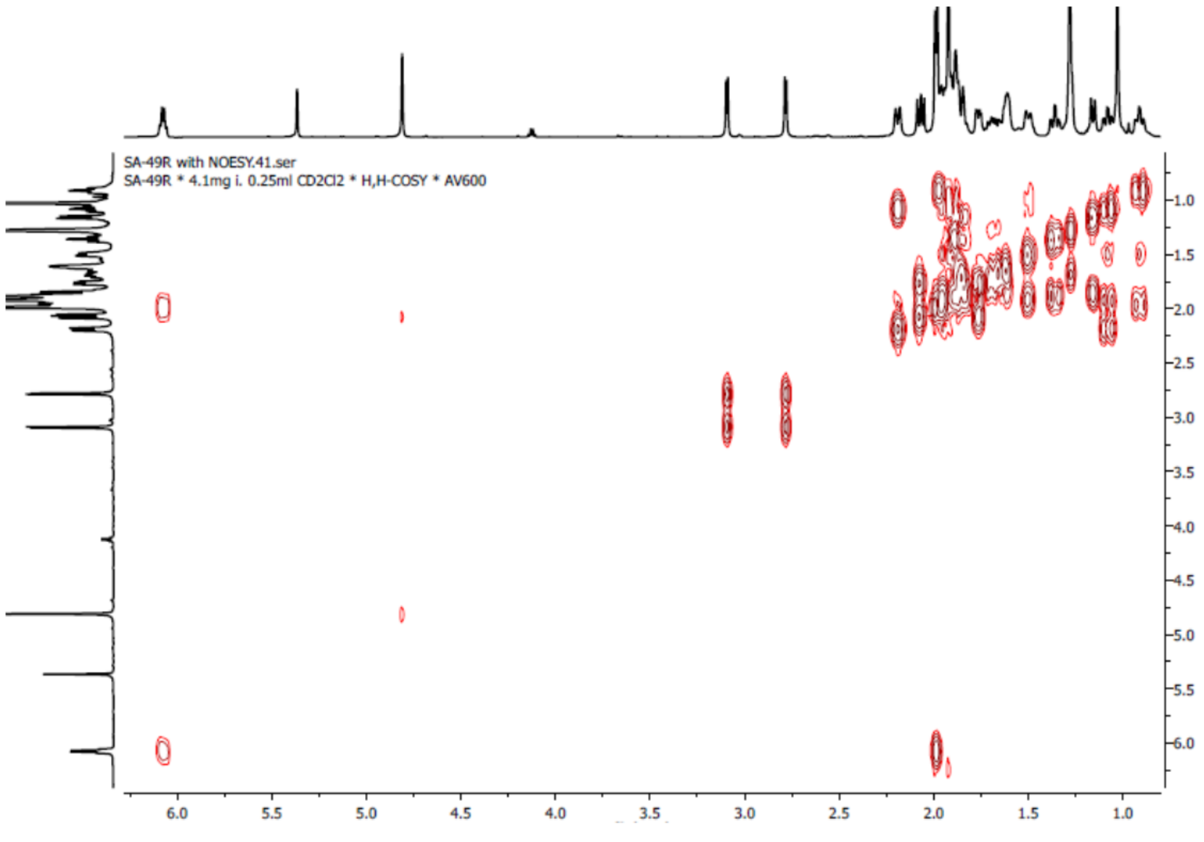

Figure S10.The ^1H-^1H COSY spectrum of 15α-angeloyloxy-16β,17-epoxy-*ent*-kauran-19-oic acid (**5**) observed at 800 MHz for $CDCl_3$ solution at 25 °C.

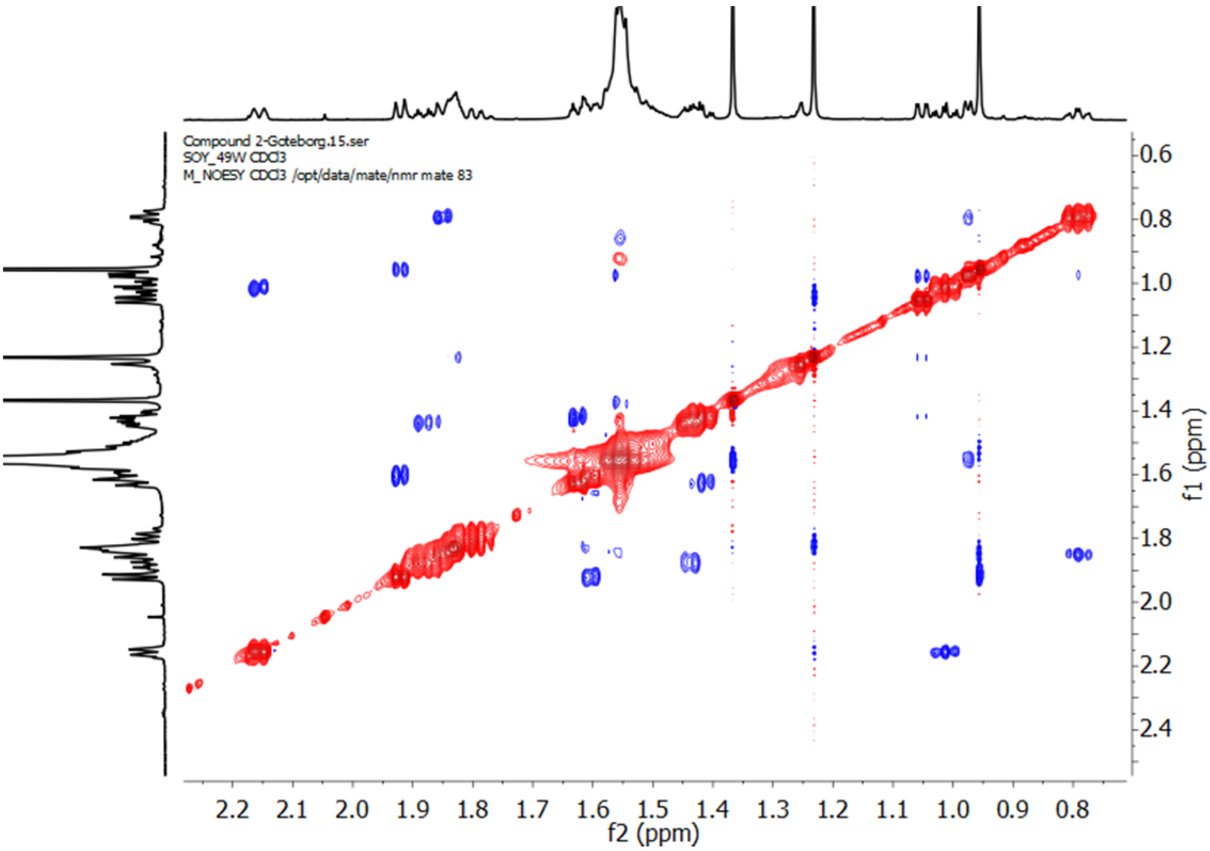

Figure S11.The ^{1}H-^{1}H NOESY NMR spectrum of 15*α*-angeloyloxy-16*β*,17-epoxy-*ent*-kauran-19-oic acid (**5**) observed at 800 MHz for $CDCl_3$ solution at 25 °C.

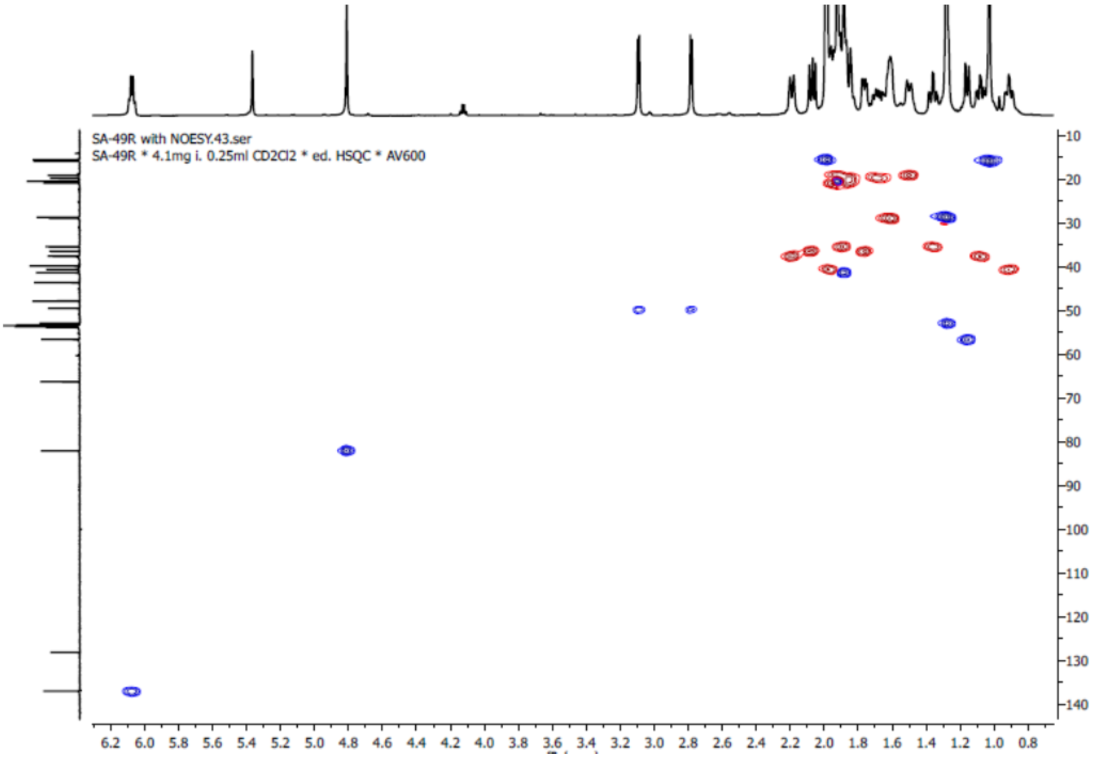

Figure S12.The ^{1}H-^{13}C HSQC NMR spectrum of 15*α*-angeloyloxy-16*β*,17-epoxy-*ent*-kauran-19-oic acid (**5**) observed at 800 and 200 MHz for $CDCl_3$ solution at 25 °C.

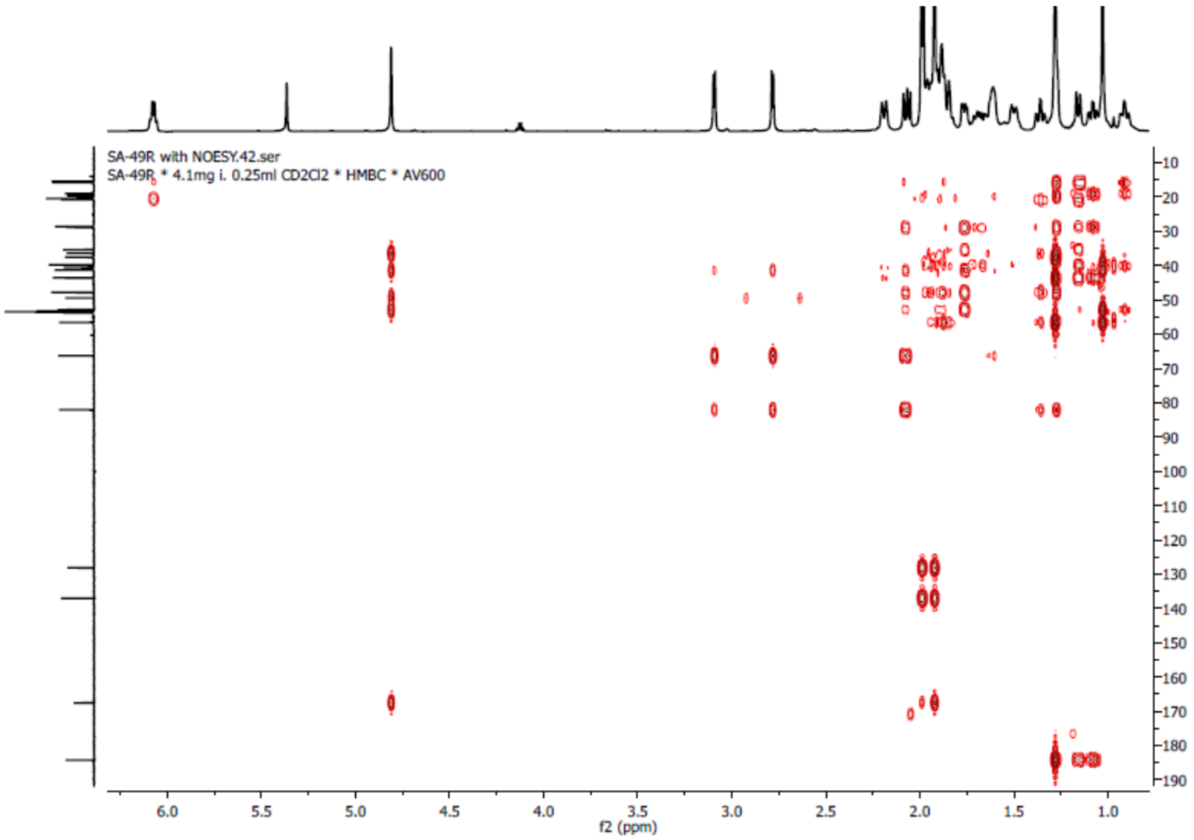

Figure S13. The ^{1}H-^{13}C HMBC NMR spectrum of 15*α*-angeloyloxy-16*β*,17-epoxy-*ent*-kauran-19-oic acid (**5**) observed at 800 and 200 MHz for $CDCl_3$ solution at 25 °C.

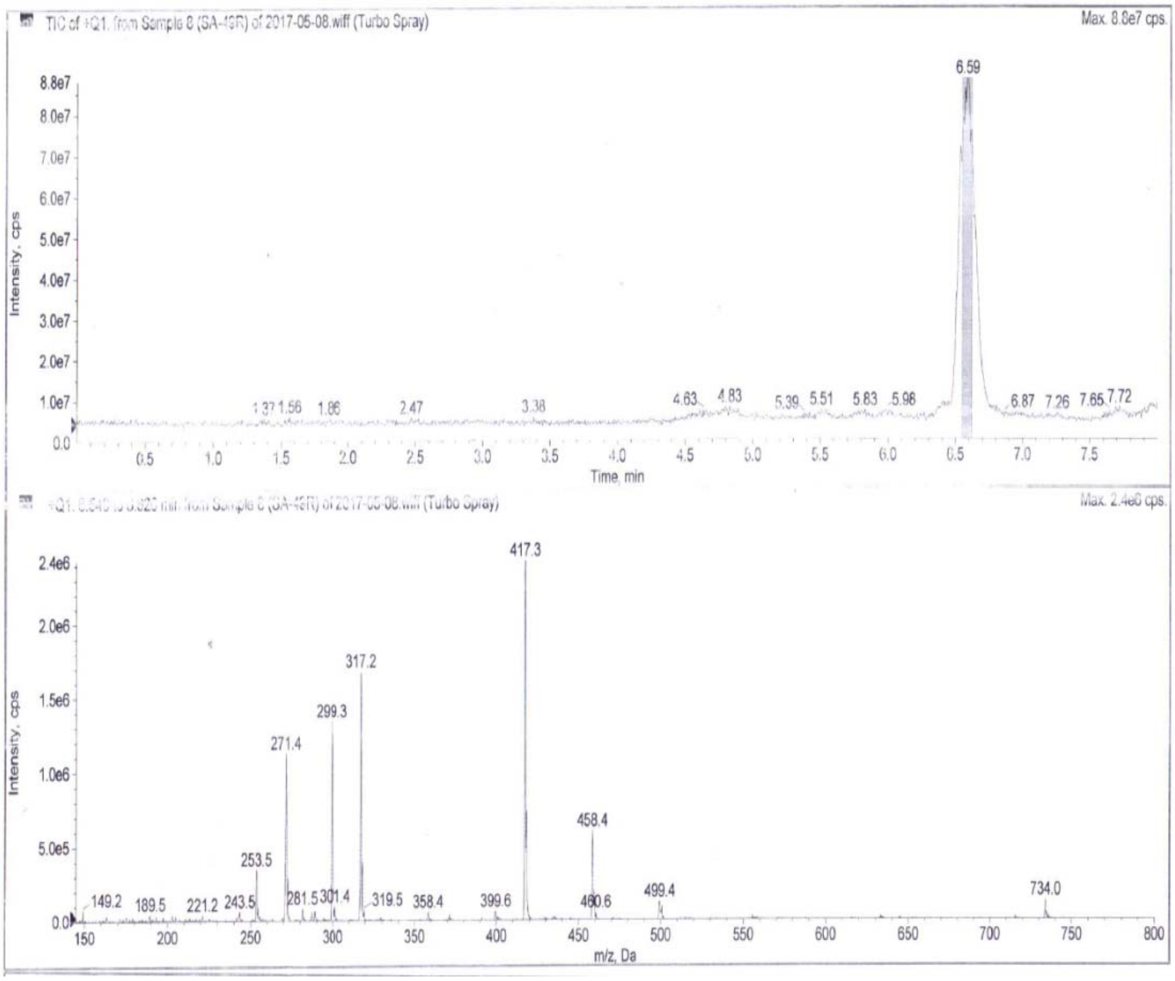

Figure S14.The ESIMS spectrum of 15α-angeloyloxy-16β,17-epoxy-*ent*-kauran-19-oic acid (**5**).

1.3. Spectroscopic data of the known compounds 2-23.

(16R) Hydroxy-ent-kauran-19-oic acid (**2**) [1,2]: Colorless crystals; mp128-130 °C; $[\alpha]_D^{20}$-102; ^{1}H NMR ($CDCl_3$, 800 MHz) δ0.79 dt (1H, *ddd*, *J*=13.2, 12.8, 4.3 Hz, CH_{2a}-1), 0.95 (3H, *s*, CH_3-20), 0.96 (1H, *d*, *J*=7.6 Hz, CH-9), 1.01 (1H, *ddd*, *J*=13.6, 13.6, 4.4 Hz, CH_{2a}-14), 1.01 (1H, *ddd*, *J*=13.6, 13.6, 4.4 Hz, CH_{2b}-14), 1.05 (1H, *dd*, *J*=11.8, 2.8 Hz, CH-5), 1.23 (3H, *s*, CH_3-18), 1.37 (3H, *s*, CH_3-17), 1.42 (1H, *ddd*, *J*=12.3, 12.0, 9.4 Hz, CH_{2a}-7), 1.43 (1H, *dddd*, *J*=13.0, 7.7, 4.1, 3.5 Hz, CH_{2a}-2), 1.50 (1H, *ddd*, *J*=8.7, 3.6, 3.1 Hz, CH_{2a}-12), 1.52 (1H, *dd*, *J*=12.6, 5.4 Hz, CH_{2a}-11), 1.55 (1H, *ddd*, *J*=7.4, 3.1, 1.8 Hz, CH_2-15), 1.56 (1H, *ddd*, *J*=7.4, 5.2, 1.8 Hz, CH_{2b}-12), 1.56 (1H, *ddd*, *J*=7.4, 5.2, 1.8, CH_{2b}-11), 1.62 (1H, *ddd*, *J*=12.7, 3.2, 3.2 Hz, CH_{2a}-3), 1.62 (1H, *ddd*, *J*=12.7, 3.2, 3.2 Hz, CH_{2b}-7), 1.79 (1H, *d*, *J*=3.1Hz, CH_{2b}-1), 1.84 (2H, *ddd*, *J*=3.1, 3.0, 2.5 Hz, CH_2-6), 1.88 (1H, *dt*, *J*=13.8, 3.8, 3.8 Hz, CH_{2b}-2), 1.92 (1H, *dd*, *J*=1.8, 1.6 Hz, CH_{2b}-3); ^{13}C NMR ($CDCl_3$, 200 MHz) δ15.5 (C-20), 18.2 (C-11), 19.0 (C-2), 22.0 (C-6), 24.4 (C-17), 26.7 (C-12), 28.9 (C-18), 37.6 (C-3), 37.9 (C-14), 39.6 (C-10), 40.6 (C-1), 41.9 (C-7), 43.5 (C-4), 45.2 (C-8), 48.8 (C-13), 55.9 (C-9), 56.8 (C-5), 57.7 (C-15), 79.3 (C-16), 180.7 (C-19); ESI-MS (30 eV) *m/z* (rel. int): [M - H_2O]$^+$ 303 (30), 285 (100), 267 (40), 205 (10), 197 (5), 149 (5); HRESI-MS (30 eV) *m/z*: 321.2429.

9β-Hydroxy-15α-angeloyloxy-ent-kaur-16-en-19-oic acid (**3**) [3]: White solid; mp 261-262°C;^{1}H NMR (CD_2Cl_2, 600 MHz) δ 1.03 (1H, *ddd*, *J*=13.2, 13.2, 4.2 Hz, CH_{2a}-14), 1.15 (3H, *s*, CH_3-20), 1.22 (3H, *s*, CH_3-18), 1.33 (1H, *ddd*, *J*=15.2, 13.4, 6.9 Hz, CH_{2a}-11), 1.48 (1H, *dddd*, *J*=3.9, 3.4, 1.5, 1.4 Hz, CH_{2a}-2), 1.50 (1H, *ddd*, *J*=3.4, 1.9, 1.5 Hz, CH_{2a}-7), 1.54 (1H, *dd*, 13.1, 1.9 Hz, CH_{2a}-1), 1.59 (1H, *dddd*, *J*=2.3, 2.2, 1.9, 1.3 Hz, CH_{2a}-3), 1.60 (1H, *m*, CH_{2a}-12), 1.70 (1H, *d*, *J*=4.9 Hz, CH_{2b}-7), 1.76 (1H, *dd*, *J*=5.7, 2.4 Hz, CH_{2b}-12), 1.80 (1H, *dd*, *J*=3.6, 3.6 Hz, CH-5), 1.87 (2H, *m*, CH_2-6), 1.87 (3H, *s*, CH_3-5'), 1.89 (1H, *ddd*, *J*=1.6, 1.5, 1.4 Hz, CH_{2b}-1), 1.96 (3H, *br s*, CH_3-4'), 1.97 (1H, *d*, *J*=1.6 Hz, CH_{2b}-2), 2.05 (1H, *d*, *J*=5.9 Hz, CH_{2b}-11), 2.12 (1H, *ddd*, *J*=13.4, 1.6, 1.6 Hz, CH_{2b}-14), 2.23 (1H, *dd*, *J*=12.1, 2.0 Hz, CH_{2b}-3), 2.78 (1H, *br s*, CH_{2a}-13), 5.09 (1H, *d*, *J*=1.4 Hz, CH_{2a}-17), 5.18 (1H, *d*, *J*=1.3 Hz, CH_{2b}-17), 6.07 (1H, *ddd*, *J*=8.7, 4.3, 2.8 Hz, CH-3'), 6.10 (1H, *s*, CH-15); ^{13}C NMR (CD_2Cl_2, 150 MHz) δ 14.9 (C-4'), 17.2 (C-20), 19.0 (C-2), 20.0 (C-5'), 21.0 (C-6), 28.5 (C-18), 28.6 (C-11), 30.0 (C-7), 32.0 (C-1), 33.8 (C-12), 37.6 (C-3), 37.6 (C-3), 37.7 (C-14), 41.3 (C-13), 43.2 (C-4), 44.1 (C-10), 49.0 (C-5), 52.8 (C-8), 75.9 (C-9), 78.8 (C-15), 109.2 (C-17), 128.0 (C-2'), 136.2 (C-3'), 156.6 (C-16), 156.6 (C-16), 167.0 (C-1'), 184.3 (C-19); ESI-MS (30 eV) *m/z* (rel. int):399 $[M-H_2O]^+$ (9), 299 (100), 253 (18), 217 (6), 203 (4), 281 (7); HRESI-MS (30 eV) *m/z*: 417.1668.

Methyl-9β-hydroxy-15α-angeloyloxy-ent-kaur-16-en-19-oate (**4**) [4]: White solid,mp 261-262°C;^{1}H NMR (CD_2Cl_2, 600 MHz)δ1.01 (3H, *s*, CH_3-18), 1.07 (1H, *dd*, *J*=4.2, 1.7 Hz, CH_{2a}-14), 1.20 (3H, *s*, CH_3-18), 1.28 (1H, *dddd*, *J*=5.3, 3.9, 2.5, 1.0 Hz, CH_{2a}-11), 1.50 (1H, *dddd*, *J*=3.7, 3.5, 2.7, 1.4 Hz, CH_{2a}-7), 1.53 (1H, *ddd*, *J*=6.5, 3.7, 2.3 Hz, CH_{2a}-2), 1.57 (1H, *ddd*, *J*=2.9, 1.3, 1.4 Hz, CH_{2a}-1), 1.60 (1H, *ddd*, *J*=2.9, 2.5, 2.4, 2.3 Hz, CH_{2a}-3), 1.63 (1H, *dd*, *J*=12.6, 2.4 Hz, CH_{2b}-7), 1.63 (1H, *m*, CH-5), 1.63 (1H, *m*, CH_{2a}-12), 1.78 (1H, *dd*, *J*=12.6, 2.4 Hz, CH_{2b}-1), 1.78 (1H, *dd*, *J*=12.6, 2.4 Hz, CH_{2b}-1), 1.82 (1H, *br s*, CH_{2a}-6), 1.88 (1H, *ddd*, *J*=2.1, 1.9, 1.3 Hz, CH_{2b}-6), 1.91 (1H, *dddd*, *J*=3.6, 3.5, 1.5, 1.4 Hz, CH_{2b}-2), 1.91 (3H, *br s*, CH_3-5'), 2.00 (3H, *d*, *J*=1.6 Hz, CH_3-4'), 2.08 (1H, *dd*, *J*=5.7, 1.4 Hz, CH_{2b}-11), 2.14 (1H, *ddt*, *J*=13.5, 3.7, 1.9, 1.9 Hz, CH_{2b}-14), 2.23 (1H, *dd*, *J*=12.3, 2.0 Hz, CH_{2b}-3), 2.82 (1H, *dd*, *J*=3.4, 1.5 Hz, CH-13), 3.66 (3H, *s*, MeO-19), 5.14 (1H, *d*, *J*=1.20 Hz, CH_{2a}-17), 5.16 (1H, *d*, *J*=1.20 Hz, CH_{2b}-17), 6.02 (1H, *br s*, CH-15), 6.04 (1H, *q*, *J*=1.7 Hz CH-3'); ^{13}C NMR ($CDCl_3$, 150 MHz) δ 15.5 (C-4'), 17.0 (C-20), 19.0 (C-2), 20.4 (C-5'), 20.9 (C-6), 28.5 (C-18), 29.1 (C-11), 30.0 (C-7), 32.0 (C-1), 33.7 (C-12), 37.6 (C-14), 37.8 (C-3), 41.2 (C-13), 44.2(C-4), 44.2 (C-10), 49.8 (C-5), 52.8 (C-8), 76.7 (C-9), 78.6 (C-15), 109.6 (C-17), 128.2 (C-2'), 137.1 (C-3'), 155.5 (C-16), 167.8 (C-1'), 177.8 (C-19), 52.8 (MeO-19); ESI-MS (30 eV), *m/z* (rel. int): 413 $[M-H_2O]^+$ (69), 313 (100), 253 (54).

15α-Angeloyloxy-ent-kaur-16α,17-epoxy-ent-kauran-19-oic acid (**5**) [5]: White solid; mp 242-243°C; ^{1}H NMR ($CDCl_3$, 800 MHz)δ0.80 (1H, *ddd*, *J*=7.2, 7.1, 1.3 Hz, CH_{2a}-1), 0.96 (1H, *ddd*, *J*=13.7, 13.7, 4.3 Hz, CH_{2a}-3), 1.03 (3H, *s*, CH_3-20), 1.16 (1H, *dd*, *J*=9.9, 4.4 Hz, CH-5), 1.25 (1H, *ddd*, *J*=14.4, 13.9, 4.4 Hz, CH_{2a}-7), 1.28 (1H, *dd*, *J*=13.8, 3.8 Hz, CH-9), 1.28 (3H, *s*, CH_3-18), 1.40 (1H, *dddd*, *J*=13.8, 3.4, 3.4, 3.1, CH_{2a}-11), 1.50 (2H,*ddd*, *J*=13.5, 7.8, 7.2 Hz, CH_2-12), 1.55 (1H, *ddd*, *J*=7.3, 3.6, 2.4 Hz, CH_{2a}-2), 1.68 (1H, *dd*, *J*=14.5, 3.3 Hz, CH_{2a}-14), 1.75 (1H, *ddd*, *J*=5.6, 5.2, 3.8 Hz, CH_{2b}-2), 1.76 (1H, *ddd*, *J*=5.7, 3.4, 2.1 Hz, CH_{2a}-6), 1.79 (1H, *ddd*, *J*=13.8, 13.2, 4.3 Hz,

CH_{2b}-7), 1.81 (1H, *dd*, *J*=13.8, 4.3 Hz, CH_{2b}-11), 1.82 (1H, *dd*, *J*=13.8, 4.4 Hz, CH-13), 1.86 (2H, *dd*, *J*=2.9, 1.4 Hz, CH_{2b}-1/ CH_{2b}-6), 1.87 (3H, *s*, CH_3-5'), 1.96 (3H, *d*, J=1.9 Hz, CH_3-4'), 1.97 (1H, *dd*, *J*=13.1, 3.4 Hz, CH_{2b}-14), 2.11 (1H, *dd*, *J*=3.7, 3.6 Hz, CH_{2b}-3), 2.78 (1H, *dd*, *J*=5.6, 1.3 Hz, CH_{2a}-17), 3.09 (1H, *dd*, *J*=5.8, 1.3 Hz, CH_{2b}-17), 4.73 (1H, *d*, *J*=1.8 Hz, CH-15), 5.96 (1H, *q*, *J*=7.1 Hz, CH-3'); ^{13}C NMR ($CDCl_3$, 200 MHz) δ 15.7 (C-4'), 15.9 (C-20), 18.9 (C-11), 19.7 (C-2), 20.6 (C-5'), 20.8 (C-6), 35.3 (C-7), 37.6 (C-3), 39.7 (C-10), 28.8 (C-12), 36.4 (C-14), 28.7 (C-18), 40.6 (C-1), 41.1 (C-13), 43.5 (C-4), 47.8 (C-8), 49.6 (C-17), 52.8 (C-9), 56.5 (C-5), 66.3 (C-16), 81.9 (C-15), 128.0 (C-2'), 137.3 (C-3'), 167.8 (C-1'), 182.7 (C-19);ESI-MS (30 eV), *m/z* (rel. int):417 $[M+H]^+$ (100), 317 (80), 299 (71), 271 (64), 253 (23).

Ent-kaur-9(11),16-dien-19-oic (**6**) [6]: White solid; mp 253-254°C; ^{1}H NMR (CD_2Cl_2, 600 MHz) δ 1.07 (3H, *s*, CH_3-20), 1.08 (1H, *ddd*, *J*=4.0, 3.9, 1.0 Hz, CH_{2a}-12), 1.28 (1H, *ddd*, *J*=1.9, 1.2, 1.1 Hz, CH_{2a}-1), 1.29 (3H, *s*, CH_3-18), 1.54 (1H, *dd*, *J*=10.8, 3.0 Hz, CH_{2a}-14), 1.55 (1H, *dd*, *J*=10.8, 3.0 Hz, CH_{2a}-2), 1.56 (1H, *dddd*, *J*=2.7,2.3, 1.7, 1.0 Hz, CH_{2a}-7), 1.65 (1H, *ddd*, *J*=10.5, 5.4, 1.1 Hz, CH_{2b}-7), 1.73 (1H, *dd*, *J*=11.1, 8.5 Hz, CH-5), 1.92 (1H, *dddd*, *J*=3.5, 3.4, 2.0, 1.9 Hz, CH_{2b}-2), 1.92 (1H, *dddd*, *J*=3.5, 3.4, 2.0, 1.9 Hz, CH2a-6), 2.01 (1H, *dd*, *J*=2.8, 1.1 Hz, CH_{2b}-1), 2.02 (1H, *dddd*, *J*=2.6, 2.2, 2.1, 1.5 Hz, CH_{2a}-3), 2.01 (1H, *ddd*, *J*=4.6, 3.4, 2.0 Hz, CH_{2b}-14), 2.18 (1H, *ddd*, *J*=2.8, 1.7, 1.5 Hz, CH_{2a}-15/ CH_{2b}-12), 2.47 (1H, *ddd*, *J*=17.1, 4.7, 2.8 Hz, CH_{2b}-3), 2.51(1H, *dddd*, *J*=2.8, 1.6, 1.5, 1.3 Hz, CH_{2b}-6), 2.67 (1H, *dd*, *J*=2.3, 1.5 Hz, CH_{2a}-15), 2.81 (1H, *ddd*, J=4.6, 2.1, 2.1 Hz, CH-13), 4.83 (1H, *dd*, *J*=2.3, 1.4 Hz, CH_{2a}-17), 4.95 (1H, *dd*, *J*=3.0, 1.5 Hz, CH_{2b}-17), 5.29 (1H, *dd*, *J*=4.1, 2.9 Hz, CH-11); ^{13}C NMR (CD_2Cl_2) δ (150 MHz, ppm):15.3 (C-20), 18.4 (C-6), 20.1 (C-2), 28.0 (C-18), 29.6 (C-14), 37.9 (C-12), 38.1 (C-3), 38.7 (C-10), 40.7 (C-1), 41.2 (C-13), 42.2 (C-8), 44.7 (C-4), 44.9 (C-7), 46.4 (C-5), 50.2 (C-15), 105.2 (C-17), 114.8 (C-11), 156.0 (C-9), 158.6 (C-16), 184.7 (C-19); ESI-MS (30 eV), *m/z* (rel. int):282 $[M-H_2O]^+$ (100), 265 (13), 247 (6).

(16S) Methoxy-ent-kaur-9(11)-en-19 oic acid (**6a**) [7]: White yellowish residue, mp 184-186 °C; ^{1}H NMR (CD_2Cl_2, 500 MHz) δ 1.02 (3H, *s*, CH_3-17), 1.05 (1H, *dd*, *J*=5.2, 3.2 Hz, CH_{2a}-3), 1.24 (3H, *s*, CH_3-18), 1.25 (1H, *dddd*, *J*=4.0, 3.6, 2.3, 1.5 Hz, CH_{2a}-7), 1.27 (3H, *s*, CH_3-20), 1.47 (1H, *ddd*, *J*=5.8, 2.7, 2.5 Hz, CH_{2a}-1), 1.48 (2H, *m*, CH_{2a}-2/ CH-12), 1.49 (1H, *m*, CH_{2a}-6), 1.65 (2H, *d*, *J*=2.6 Hz, CH-15), 1.75 (1H, *dd*, *J*=12.2, 8.7 Hz, CH-5), 1.91 (1H, *ddd*, *J*=6.5, 6.3, 3.9 Hz, CH_{2b}-1), 1.92 (1H, *dd*, *J*=16.5, 6.9 Hz, CH_{2b}-2), 1.98 (1H, *dd*, *J*=10.1, 3.5 Hz, CH_{2b}-7), 2.05 (1H, *dd*, *J*=4.3, 1.7 Hz, CH_{2a}-14), 2.15 (1H, *ddd*, *J*=3.5, 3.2, 2.1 Hz, CH_{2b}-3), 2.22 (1H, *dd*, *J*=5.3, 2.3 Hz, CH-13), 2.28 (1H, *dd*, *J*=2.6, 2.6 Hz, CH_{2b}-14), 5.21 (1H, *dd*, *J*=1.9, 1.7 Hz, CH-11), 3.16 (3H, *s*, 16-OCH_3); ^{13}C NMR (CD_2Cl_2, 125 MHz) δ 18.4 (C-2), 20.2 (C-6), 21.5 (C-20), 23.3 (C-17), 28.0 (C-18), 30.2 (C-12), 30.9 (C-14), 38.2 (C-3), 38.7 (C-10), 41.0 (C-7), 41.7 (C-13), 42.6 (C-4), 43.4 (C-1), 44.6 (C-8), 46.5 (C-15), 56.0 (C-5), 87.2 (C-16), 114.2 (C-11), 157.5 (C-9), 178.2 (C-19), 49.4 (MeO-16); ESI-MS (30 eV), *m/z* (rel. int): 347 $[M+H]^+$ (100), 315 (34), 269 (21), 287 (4), 313 (3), 229 (2), 193 (2), 149 (2); HRESI-MS (30 eV), *m/z* (rel. int): 347.2586.

(16R) Ent-kaur-9(11)-en-19-oic acid (**6b**): White yellowish residue; mp 128-130 °C; ^{1}H NMR (CD_2Cl_2, 500 MHz) δ 1.02 (3H, *s*, CH_3-20), 1.06 (3H, *d*, *J*=8.3 Hz, CH_3-17), 1.09 (1H, *dd*, *J*=4.3,

4.0 Hz, CH_{2a}-3), 1.26 (3H, *s*, CH_3-18), 1.28 (1H, *dd*, *J*=5.4, 5.0 Hz, CH_{2a}-1), 1.39 (1H, *dd*, J=2.2, 1.6 Hz, CH_{2b}-14), 1.40 (1H, *dd*, *J*=7.1, 1.5 Hz, CH_{2a}-15), 1.43 (1H, *dd*, *J*=11.1, 3.7 Hz, CH_{2a}-12), 1.44 (2H, *ddd*, *J*=9.3, 7.1, 3.6 Hz, CH_2-2), 1.50 (1H, *ddd*, *J*=3.8, 3.2, 3.2 Hz, CH-13), 1.53 (1H, *ddd*, *J*=14.1, 3.5, 3.5 Hz, CH_{2a}-6), 1.73 (1H, *dd*, *J*=3.9, 1.8 Hz, CH-5), 1.82 (1H, *dd*, *J*=6.6, 4.2 Hz, CH_{2b}-15), 1.92 (1H, *ddd*, *J*=3.9, 3.5, 1.3 Hz, CH_{2b}-6), 1.98 (1H, *ddd*, *J*=2.1, 1.8, 1.6 Hz, CH_{2b}-1), 2.16 (1H, *dddd*, *J*=7.1, 3.7, 2.9, 1.5 Hz, CH_{2b}-3), 2.17 (2H, *dd*, *J*=2.7, 1.5 Hz, CH_2-7), 2.36 (1H, *dd*, *J*=3.0, 2.1 Hz, CH_{2a}-14), 2.44 (1H, *ddddd*, *J*=2.0, 1.5, 1.5, 1.4, 1.2 Hz, CH-16), 5.24 (1H, *dd*, *J*=3.5, 3.1 Hz, CH-11); ^{13}C NMR (CD_2Cl_2, 125 MHz) δ 18.5 (C-20),18.6 (C-2), 20.2 (C-6), 23.2 (C-17), 28.0 (C-19), 29.8 (C-7), 30.2 (C-12), 37.1(C-16), 37.8 (C-14), 38.2 (C-3), 38.7 (C-10), 41.1 (C-1), 42.4 (C-4), 44.7 (C-8), 46.0 (C-13), 46.6 (C-5), 49.8 (C-15), 115.0 (C-11), 158.3 (C-9), 184.8 (C-18); ESI-MS (30 eV), *m/z* (rel. int): 303 $[M+H]^+$ (100), 257 (19), 287 (6), 241 (3), 175 (2); HRESI-MS (30 eV), *m/z* (rel. int):303.2324.

15α-Angeloyloxy-ent-kaur-16-en-19-oic acid (**7**) [8]: White solid; mp 256-257°C; ^{1}H NMR (CD_2Cl_2, 600 MHz) δ 0.91 (1H, *d*, *J*=4.2 Hz, CH_{2a}-1), 1.01 (3H, *s*, CH_3-20), 1.08 (1H, *d*, *J*=4.2 Hz, CH_{2a}-3), 1.17 (1H, *dd*, *J*=2.3, 1.3 Hz, CH-5), 1.27 (3H, *s*, CH_3-18), 1.31 (1H, *dd*, *J*=10.3, 2.80 Hz, CH_{2a}-7), 1.32 (1H, *dd*, *J*=3.8, 2.2 Hz CH-9), 1.50 (1H, *dd*, *J*=2.9, 1.7 Hz, CH_{2a}-14), 1.55 (1H, *dd*, *J*=1.9, 1.8 Hz, CH_{2a}-2), 1.68 (2H, *d*, *J*=10.6 Hz, CH_2-11), 1.69 (2H, *m*, CH_{2b}-12/CH_{2b}-7), 1.80 (1H, *dd*, *J*=1.8, 1.7 Hz, CH-6), 1.87 (3H, *s*, CH_3-5'), 1.91 (1H, *d*, *J*=1.5 Hz, CH_{2b}-2), 1.93 (1H, *dd*, *J*=3.1, 2.3 Hz, CH_{2b}-1), 1.91 (3H, *d*, *J*=1.6 Hz, CH-4'), 2.04 (1H, *d*, *J*=1.0 Hz, CH_{2b}-14), 2.18 (1H, *d*, *J*=13.4 Hz, CH_{2b}-3), 2.83 (1H, *dd*, *J*=4.7, 2.8 Hz, CH-13), 5.14 (1H, *d*, *J*=1.2 Hz, CH_{2a}-17), 5.17 (1H, *d*, *J*=1.2 Hz, CH_{2b}-17), 5.37 (1H, *br s*, CH-15), 6.08 (1H, *dddd*, *J*=8.7, 7.2, 5.7, 1.5 Hz, CH-3'); ^{13}C NMR (CD_2Cl_2, 150 MHz) δ 15.5 (C-4'), 15.6 (C-20), 18.4 (C-11), 19.0 (C-2), 20.4 (C-5'), 20.8 (C-6), 28.7 (C-18),32.6 (C-12), 35.0 (C-7), 37.3 (C-14),37.6 (C-3), 39.8 (C-10), 40.5 (C-1), 42.6 (C-13), 43.6 (C-4), 47.5 (C-8), 52.9 (C-9),56.5 (C-5), 82.4 (C-15), 109.5 (C-17), 128.3 (C-2'), 136.9 (C-3'), 156.0 (C-16), 167.7 (C-1'), 183.7 (C-19); ESI-MS (30 eV), *m/z* (rel. int): 401 $[M+H]^+$ (7), 301 (100), 371 (8), 313 (11), 255 (13).

(16S) Ent-kauran-19-oic acid (**8**) [9]: Colorless crystals; ^{1}H NMR (Acetone-d_6, 500 MHz) δ 0.85 (1H, *ddd*, *J*=4.8, 4.6, 2.9 Hz, CH_{2a}-1), 0.99 (1H, *dd*, *J*=2.0, 1.9 Hz, CH_{2a}-15), 1.01 (3H, *s*, CH_3-20), 1.01 (1H, *br s*, CH-9), 1.03 (3H, *d*, *J*=8.6 Hz, CH_3-17), 1.04, (1H, *dd*, *J*=2.5, 1.0 Hz, CH_{2a}-3), 1.05 (1H, *ddd*, *J*=4.1, 4.0, 3.3 Hz, CH_{2b}-1), 1.09 (1H, *dd*, *J*=4.1, 2.4 Hz CH-5), 1.20 (3H, *s*, CH_3-18), 1.41 (1H, *dddd*, *J*=2.5, 2.4, 1.6, 1.5 Hz, CH_2-11), 1.46 (1H, *ddd*, *J*=2.6, 2.3, 1.4 Hz, CH_{2a}-2/CH_{2a}-12), 1.47 (1H, *J*=2.6, 2.3, 1.4 Hz, CH_{2a}-14), 1.52 (1H, *ddd*, *J*=6.1, 3.3, 3.2 Hz, CH_{2a}-6), 1.56 (1H, *ddd*, *J*=4.1, 3.2, 2.6 Hz, CH_{2b}-6), 1.58 (1H, *ddd*, *J*=4.1, 3.2, 2.6 Hz, CH_{2b}-14), 1.64 (1H, *ddd*, 6.3, 1.2, 1.0 Hz, CH_{2b}-12), 1.65 (2H, *m*, CH_{2b}-2/CH_{2b}-15), 1.85 (1H, *ddd*, *J*=11.6, 3.3, 3.3 Hz, CH_{2a}-7), 1.85 (1H, *ddd*, *J*=3.2, 1.8, 1.6 Hz, CH-16), 2.01 (1H, *ddd*, *J*=3.7, 1.2, 1.2 Hz, CH_{2b}-7), 2.14 (1H, *ddd*, *J*=2.1, 1.7, 1.2 HZ, CH_{2b}-3); ^{13}C NMR (Acetone-d_6, 125 MHz) δ15.2 (C-20), 15.3 (C-17), 18.7 (C-11), 19.1 (C-2), 22.2 (C-6), 25.7 (C-12), 28.4 (C-19), 34.3 (C-4), 38.0 (C-3), 39.5 (C-10), 40.0 (C-7), 40.6 (C-1), 40.7 (C-14), 42.1 (C-16), 43.1 (C-13), 44.7 (C-8), 48.7 (C-15), 56.5 (C-9), 56.7 (C-5),

178.2 (C-18); ESI-MS (30 eV), *m/z* (rel. int):305 $[M+H]^+$ (72), 282 (100), 259 (42), 247 (23), 191 (13), 287 (11), 281 (10), 149 (19).

Ent-kaur-9(11),16-dien-12-one (**9**) [10]: White solid; ^1H NMR (Acetone-d_6, 600 MHz) δ 1.20 (3H, *s*, CH_3-19), 1.28 (3H, *s*, CH_3-18), 1.32 (3H, *s*, CH_3-20), 1.32 (1H, *ddd*, *J*=14.4, 4.0, 3.1 Hz, CH_{2a}-1), 1.56 (1H, *dddd*, *J*=4.3, 4.1, 3.7, 3.4 Hz, CH_{2a}-2), 1.84 (1H, *d*, *J*=4.7 Hz, CH-15), 1.92 (1H, *dd*, *J*=11.2, 2.6 Hz, CH-15), 1.92 (1H, *dd*, *J*=11.2, 2.6 Hz, CH_{2a}-3), 2.00 (1H, *ddd*, *J*=2.7, 2.6, 1.7 Hz, CH_{2b}-2), 2.01 (1H, *ddd*, *J*=2.6, 2.6, 1.6 Hz, CH_{2a}-6), 2.02 (1H, *ddd*, *J*=5.2, 4.2, 2.7 Hz, CH_{2b}-1), 2.47 (2H, *dd*, *J*=2.6, 2.6 Hz, CH-5/CH_{2a}-14), 2.62 (1H, *m*, CH_{2b}-6/CH_{2b}-14), 3.29 (1H, *dd*, *J*=5.7, 2.1 Hz CH-13), 4.95 (1H, *d*, *J*=1.2 Hz, CH-17), 5.13 (1H, *d*, *J*=1.3 Hz, CH-17), 5.67 (1H, *s*, CH-11); ^{13}C NMR (Acetone-d_6, 150 MHz) δ 18.1 (C-6), 19.9 (C-2), 22.3 (C-18), 27.5 (C-3), 28.3 (C-20), 28.6 (C-19), 28.7 (C-7), 37.9 (C-8), 39.7 (C-1), 40.1 (C-4), 43.9 (C-14), 44.6 (C-5), 45.3 (C-10), 48.1 (C-15), 58.2 (C-13), 109.6 (C-17), 119.4 (C-11), 147.9 (C-16), 180.7 (C-9), 197.8 (C-12).

Methyl-ent-kaur-16-en-19-oate (**10**) [11]. White solid; mp 261-262°C;^1H NMR (Acetone-d_6, 600 MHz) δ 0.89 (1H, *ddd*, *J*=4.4, 2.1, 1.3, 1.2 Hz, CH-13), 1.00 (3H, *s*, CH_3-20), 1.07 (1H, *ddd*, *J*=2.7, 2.2, 1.0 Hz, CH_{2a}-3), 1.12 (1H, *dd*, *J*=5.0, 1.8 Hz, CH-9), 1.18 (3H, *s*, CH_3-18), 1.31 (2H, *d*, J=3.2 Hz, CH_2-14), 1.40 (1H, *dddd*, *J*=2.7, 1.9, 1.7, 1.6 Hz, CH_{2a}-2), 1.53 (1H, *d*, *J*=3.8 Hz, CH_{2a}-1), 1.54 (1H, *ddd*, *J*=3.6, 3.5, 1.4 Hz, CH_{2a}-12), 1.64 (2H, *ddd*, *J*=2.3, 1.4, 1.0 Hz, CH_2-11) 1.90 (2H, *m*, CH_{2b}-1/CH_{2b}-2), 1.91 (1H, *ddd*, *J*=6.5, 3.5, 1.8 Hz, CH_2-6), 2.06 (1H, *ddd*, *J*=2.8, 2.8, 2.8 Hz, CH_{2b}-12), 2.15 (1H, *ddd*, *J*=1.9, 1.7, 1.2 Hz, CH_{2b}-3), 2.16 (1H, *dd*, *J*=1.7, 1.4 Hz, CH_2-14), 3.33 (3H, *s*, MeO-19); ^{13}C NMR (Acetone-d_6, 150 MHz) δ15.3 (C-20), 18.2 (C-11), 19.1 (C-2), 21.8 (C-6), 28.4 (C-12), 28.6 (C-18),32.9 (C-14), 37.9 (C-3), 39.5 (C-10), 40.6 (C-1), 41.2 (C-13), 43.1 (C-7), 43.8 (C-4), 44.1 (C-8), 48.8 (C-15), 49.9 (MeO-19), 55.0 (C-9), 56.6 (C-5), 102.7(C-17), 155.4 (C-16), 178.2 (C-19); ESI-MS (30 eV), *m/z* (rel. int): 317 $[M+H]^+$ (100), 251 (87), 297 (51), 175 (58), 205 (43), 149 (69), 259 (34).

Ent-kaur-16-en-19-oic acid (**11**) [6,12]. White solid ;mp 247-248°C; ^1H NMR ($CDCl_3$, 800 MHz) δ 1.00 (3H, *s*, CH_3-20), 1.03 (1H, *d*, *J*=4.2 Hz, CH_{2a}-3), 1.06 (2H, *ddd*, *J*=2.8, 1.7, 1.6 Hz, CH_2-12), 1.11 (1H, *dd*, J=7.1, 6.5 Hz, CH_{2a}-1), 1.12 (2H, *m*, CH-5/CH-9), 1.21 (3H, *s*, CH_3-18), 1.41 (1H, *ddd*, J=6.3, 4.9, 2.1 Hz, CH_{2a}-2), 1.49 (1H, *ddd*, J=4.6, 3.4, 2.1 Hz, CH_{2a}-7), 1.64 (1H, *dd*, J=3.1, 2.0 Hz, CH_{2a}-11), 1.76 (1H, *dd*, *J*=11.7, 4.7 Hz, CH_{2a}-14), 1.84 (1H, *ddd*, J=4.8, 3.8, 2.2 Hz, CH_{2b}-1), 1.90 (1H, *dd*, J=2.4, 2.3 Hz, CH_{2b}-2), 1.91 (2H, *m*, CH_2-6), 1.96 (1H, *dd*, *J*=2.7, 2.6 Hz, CH-13), 2.02 (1H, *ddd*, *J*=2.3, 2.1, 2.0 Hz, CH_{2a}-15), 2.06 (1H, *d*, *J*=10.0 Hz, CH_{2a}-14), 2.07 (2H, *m*, CH_{2b}-7/CH_{2b}-15), 2.15 (1H, *ddd*, *J*=2.9, 2.3, 1.3 Hz, CH_{2b}-3), 2.63 (1H, *dd*, *J*=4.2, 4.2 Hz, CH_{2b}-11), 4.78 (1H, *d*, *J*=1.2 Hz, CH_{2a}-17), 4.91 (1H, *d*, *J*=2.0 Hz, CH_{2b}-17); ^{13}C NMR ($CDCl_3$, 200 MHz) δ15.3 (C-20), 18.2 (C-11), 19.1 (C-2), 21.8 (C-6), 28.4 (C-12), 28.6 (C-18), 28.7 (C-7), 32.9 (C-14), 37.9 (C-3), 39.5 (C-10), 40.6 (C-1), 43.1 (C-4), 43.8 (C-13), 44.1 (C-8), 48.8 (C-15), 55.0 (C-9), 56.6 (C-5), 102.9 (C-17), 155.4 (C-16), 178.2 (C-19); ESI-MS (30 eV), *m/z* (rel. int): 285 $[M-H_2O]^+$ (100), 265 (19), 247.3 (8), 212 (4).

Ent-kaur-16-en-19-ol (**12**) [13]: ^{1}H NMR ($CDCl_3$, 800 MHz) δ 0.73 (1H, *ddd*, *J*=3.9, 3.8, 3.8 Hz, CH_{2a}-1), 0.85 (1H, *ddd*, *J*=2.7, 1.2, 1.2 Hz, CH-5), 0.89 (1H, *s*, CH_3-18), 0.94 (1H, *s*, CH_3-20), 1.02 (2H, *m*, CH-9/CH_{2a}-14), 1.12 (2H, *dd*, *J*=7.4, 4.7 Hz, CH-12), 1.28 (2H, *ddd*, *J*=4.4, 2.6, 1.3 Hz, CH_{2a}-2), 1.27 (1H, *ddd*, *J*=4.4, 2.6, 1.3 Hz, CH_{2a}-11), 1.35 (1H, *dddd*, *J*=3.9, 3.7, 3.6, 3.6 Hz, CH_{2b}-11), 1.40 (1H, *ddd*, *J*=4.1, 3.1, 1.8 Hz, CH_{2a}-3), 1.50 (1H, *ddd*, *J*=5.8, 4.9, 3.0 Hz, CH_{2b}-6), 1.52 (1H, *m*, CH_{2b}-2/CH_{2b}-3), 1.57 (1H, *dd*, *J*=2.9, 1.8 Hz, CH_{2b}-14), 1.70 (1H, *ddd*, *J*=1.8, 1.7, 1.3 Hz, CH_{2b}-1), 1.73 (1H, *ddd*, *J*=1.7, 1.7, 1.2 Hz, CH_{2a}-7), 1.80 (1H, *ddd*, *J*=3.4, 1.7, 1.7 Hz, CH_{2b}-7), 1.91 (1H, *d*, *J*=2.5 Hz, CH_{2a}-15), 1.99 (1H, *d*, *J*=5.7 Hz, CH_{2b}-15), 2.57 (1H, *dd*, *J*=10.7, 3.3, 3.3 Hz, CH-15), 3.38 (1H, *dd*, *J*=10.9, 1.2 Hz, CH_{2a}-19), 3.68 (1H, *d*, *J*=10.9 Hz, CH_{2b}-19), 4.66 (1H, *d*, *J*=2.2 Hz, CH_{2a}-17), 4.72 (1H, *d*, *J*=1.7 Hz, CH_{2b}-17); ^{13}C NMR ($CDCL_3$, 200 MHz) δ 18.1 (C-20), 18.2 (C-11), 18.3 (C-6), 20.5 (C-2), 27.1 (C-18), 29.7 (C-12), 33.1 (C-3), 35.6 (C-4),38.7 (C-14),39.7 (C-10), 40.4 (C-1), 41.6 (C-7), 44.0 (C-13), 44.2 (C-8), 49.1 (C-15), 56.2 (C-9),56.8 (C-5),65.6 (C-19), 102.9 (C-17), 155.9 (C-16).

Lanosterol (**13**) [14]: White solid ; mp 137-138 °C; ^{1}H NMR (Acetone-d_6, 600 MHz) δ0.80 (3H, *s*, CH-18), 0.90 (3H, *s*, CH-28), 0.94 (6H, *br s*, CH-21/CH-30), 1.01 (3H, *s*, CH-29), 1.06 (3H, *s*, CH-19), 1.15 (1H, *ddd*, *J*=3.6, 2.7, 1.9 Hz, CH-5), 1.57 (1H, *ddddd*, *J*=8.5, 8.3, 4.1, 3.0, 2.9 Hz, CH-20), 1.61 (1H, *dd*, *J*=8.5, 4.1 Hz, CH-17), 1.62 (3H, *s*, CH-27), 1.68 (1H, *dd*, *J*=3.5, 2.4 Hz, CH_{2a}-12), 1.73 (1H, *br s*, CH_{2b}-12), 1.74 (3H, *s*, CH-26), 3.19 (1H, *dd*, *J*=5.7, 5.6 Hz, CH-3), 5.14 (3H, *br s*, CH-24); ^{13}C NMR (Acetone-d_6, 150 MHz) δ15.1 (C-18), 15.3 (C-29),16.8 (C-27), 18.3 (C-21), 18.8 (C-6), 19.7 (C-19), 21.3 (C-11), 23.9 (C-30), 24.5 (C-23), 27.5 (C-7), 27.7 (C-26), 27.9 (C-28), 28.5 (C-2),28.7 (C-16),29.5 (C-15),30.9 (C-12),35.2 (C-22),35.3 (C-20), 35.7 (C-1), 37.1 (C-4), 37.1 (C-10), 44.0 (C-13), 49.6 (C-14), 50.2 (C-17), 51.1 (C-5), 77.6 (C-3), 124.0 (C-24), 133.3 (C-9), 134.3 (C-8/C-25).

Stigmasta-5,22(E)-dien-3β-ol (**14**) [15]: White solid; ^{1}H NMR (CD_2Cl_2) δ (800 MHz, 600Mhz, ppm): 0.75 (3H, *d*, *J*=6.4 Hz, CH_3-26), 0.85 (3H, *s*, CH_3-18), 0.89 (3H, *d*, *J*=2.2 H, CH_3-29), 0.97 (3H, *s*, CH_3-19), 0.98 (1H, *d*, *J*=6.5 Hz, CH-9), 1.05 (2H, *m*, CH_{2a}-1/CH-14/ CH_2-21), 1.06 (3H, *br d*, CH_2-21), 1.07 (2H, *s*, CH_2-28), 1.21 (2H, *m*, CH-25/CH-17), 1.28 (2H, *m*, CH-16), 1.31 (3H, *brs*, CH_3-27), 1.57 (2H, *m*, CH_2-11), 1.58 (2H, *ddd*, *J*=6.8, 6.0, 3.2 Hz, CH-15), 1.57 (1H, *m*, CH-24), 1.83 (2H, *ddd*, *J*=6.3, 3.7, 3.5 Hz, CH_2-2), 1.77 (2H, *dd*, *J*=3.7, 3.4 Hz, CH_2-7), 1.89 (1H, *dt*, *J*=13.2, 3.4, 3.4 Hz, CH_{2b}-1), 2.03 (3H, *m*, CH-8/CH_{2b}-12/CH-20), 2.21 (1H, *m*, CH_{2a}-12), 3.38 (1H, *ddd*, *J*=6.3, 5.4, 4.2 hz, CH-3), 5.15 (1H, *m*, CH-23), 5.20 (1H, *m*, CH-22), 5.39 (1H, *dd*, *J*=5.1, 2.6 Hz, CH-6); ^{13}C NMR (CD_2Cl_2, 200 MHz) δ 11.8 (C-27),12.0 (C-18), 18.7 (C-19), 19.1 (C-30), 20.8 (C-29), 21.0 (C-11), 21.0 (C-21), 24.3 (C-15), 25.4 (C-26), 29.6 (C-16), 29.7 (C-28), 31.7 (C-2), 31.9 (C-8), 31.9 (C-7), 36.4 (C-10), 37.2 (C-1), 39.5 (C-12), 40.5 (C-20), 42.1(C-13), 42.3 (C-4), 51.2 (C-24), 55.9 (C-17) 56.8 (C-14), 71.6 (C-3), 121.4 (C-6), 129.2 (C-23), 138.4 (C-22), 140.9 (C-5).

3-hydroxy-Olean-12-en-29-oic acid (**15**) [16]: White solid; ^{1}H NMR (DMSO-d_6, 600 MHz) δ0.69 (3H, *s*, CH_3-26), 0.70 (3H, *s*, CH_3-28), 0.78 (1H, *dd*, *J*=5.6, 4.1 Hz, CH-18), 0.85 (3H, *s*, CH_3-27),

0.88 (3H, *s*, CH_3-25), 0.89 (3H, *s*, CH_3-23), 0.95 (1H, *ddd*, *J*=4.8, 3.6, 3.1 Hz, CH_{2a}-1), 0.96 (1H, *m*, CH-5), 1.10 (3H, *s*, CH_3-24), 1.23 (3H, *s*, CH_3-30), 1.25 (1H, *dd*, *J*=7.7, 3.3 Hz, CH_{2a}-11), 1.34 (1H, *dd*, *J*=6.6, 4.7 Hz, CH_{2a}-19), 1.41 (2H, *m*, CH_{2b}-2/CH_{2a}-15), 1.42 (2H, *dd*, *J*=9.8, 5.2 Hz, CH_2-22), 1.46 (3H, *m*, CH_{2b}-15/ CH_{2b}-19/ CH_{2a}-21), 1.47 (1H, *ddd*, *J*=12.8, 11.7, 5.2 Hz, CH_{2b}-1), 1.48 (2H, *ddd*, *J*=12.8, 11.7, 5.2 Hz, CH-6), 1.57 (1H, *ddd*, *J*=8.6, 5.8, 5.3 Hz, CH_{2a}-7), 1.71 (1H, *m*, CH_{2b}-7/ CH-9), 1.72 (2H, *dd*, *J*=10.3, 3.1 Hz, CH_2-16), 1.80 (1H, *dd*, *J*=12.3, 7.6 Hz, CH_{2b}-11), 2.00 (1H, *dd*, *J*=8.7, 3.7 Hz, CH_{2b}-21), 2.95 (1H, *dd*, *J*=12.1, 4.0 Hz, CH-3), 3.00 (1H, *dd*, *J*=5.4, 4.8 Hz, CH-12); ^{13}C NMR (DMSO-*d*$_6$, 150 MHz) δ15.7 (C-25), 15.8 (C-27/C-24), 16.5 (C-26), 17.3 (C-28), 18.4 (C-6), 19.3 (C-11), 22.4 (C-16), 24.9 (C-30), 26.9 (C-15/C-22), 28.7 (C-20), 29.1 (C-23), 30.7 (C-7), 33.4 (C-2), 37.1 (C-10), 37.5 (C-21), 38.1 (C-8), 38.8 (C-1),40.5 (C-4), 41.5 (C-18), 42.2 (C-14), 43.3 (C-19), 45.1 (C-17), 48.2 (C-9), 56.4 (C-5), 77.3 (C-3), 121.7 (C-12), 144.5 (C-13), 179.1 (C-29).

Carissone (**16**) [17]. White solid; ^{1}H NMR (Acetone-*d*$_6$, 600 MHz) δ1.18 (3H, *s*, CH_3-12), 1.23 (3H, *s*, CH_3-13), 1.42 (3H, *s*, CH_3-15), 1.68 (3H, *s*, CH_3-14), 1.73 (1H, *dd*, *J*=5..4, 3.4 Hz, CH_{2a}-1), 1.87 (1H, *dd*, *J*=5.2, 1.3 Hz, CH_{2a}-6), 1.94 (1H, *dd*, *J*=3.2, 1.7 Hz, CH_{2b}-1), 1.98 (1H, *dd*, *J*=4.5, 3.9 Hz, CH_{2b}-6), 2.05 (1H, *d*, *J*=2.9 Hz, CH-7), 2.27 (1H, *dd*, *J*=11.2, 4.5 Hz, CH_{2a}-2), 2.35 (1H, *m*, CH_{2b}-2/CH_{2a}-8), 2.65 (1H, *dd*, *J*=7.4, 6.8 Hz, CH_{2b}-8); ^{13}C NMR (Acetone-*d*$_6$, 150 MHz) δ9.9 (C-14), 23.9 (C-15), 25.2 (C-8), 27.1 (C-12), 28.7 (C-13), 34.4 (C-6), 35.4 (C-2),42.7 (C-10),46.5 (C-9), 48.2 (C-7),53.9 (C-1), 78.9 (C-11), 125.3 (C-4), 163.0 (C-5), 196.9 (C-3).

Methyl-15α-angeloyloxy-ent-kaur-16-en-19-oate (**17**) [11,18]. Colorless crystals; ^{1}H NMR (CD_2Cl_2, 500 MHz) δ0.88 (3H, *s*, CH_{2a}-20), 0.92 (1H, *ddd*, *J*=13.6, 6.9, 6.9 Hz CH_{2a}-1), 1.06 (1H, *dd*, *J*=13.7, 4.1 Hz, CH_{2a}-3) 1.07 (1H, *dd*, *J*=12.2, 8.7 Hz, CH_{2a}-6), 1.10 (1H, *dd*, *J*=7.5, 6.5 Hz, CH-5), 1.17 (1H, *dd*, *J*=11.9, 7.2 Hz, CH_{2b}-3), 1.18 (3H, *s*, CH_{2a}-18), 1.31 (1H, *dd*, *J*=13.2, 3.4 Hz, CH_{2a}-7), 1.47 (2H, *m*, CH_{2a}-2/CH_{2a}-12), 1.53 (2H, *m*, CH_{2b}-11/CH_{2a}-14), 1.60 (1H, *ddd*, *J*=14.3, 5.6, 5.6 Hz, CH_{2b}-12), 1.66 (2H, *m*, CH_{2b}-2/CH_{2a}-11), 1.70 (1H, *ddd*, *J*=6.2, 3.5, 3.5 Hz, CH_{2b}-7) 1.90 (2H, *m*, CH_{2b}-1/CH_{2b}-6), 1.91 (3H, *br s*, CH-5'), 1.99 (3H, *d*, *J*=6.9 Hz, CH-4'), 2.02 (1H, *dd*, *J*=13.8, 1.5 Hz, CH_{2b}-14), 2.82 (1H, *br s*, CH-13), 3.64 (3H, *s*, MeO-19), 5.29 (1H, *br s*, CH-15), 5.11 (1H, *d*, *J*=1.4 Hz, CH_{2a}-17), 5.15 (1H, *d*, *J*=1.3 Hz, CH_{2b}-17), 6.07 (1H, *dddd*, *J*= 8.7, 7.2, 5.8, 1.5 Hz, CH-3'); ^{13}C NMR (CD_2Cl_2, 125 MHz) δ15.5 (C-20), 15.0 (C-4'), 19.1 (C-2), 18.4 (C-11), 20.5 (C-5'), 20.9 (C-6), 28.4 (C-18), 32.7 (C-12), 35.1 (C-7), 37.3 (C-14), 37.9 (C-3), 39.6 (C-10), 40.6 (C-1), 42.7 (C-13), 43.6 (C-4), 47.5 (C-8), 50.9 (MeO-19), 53.0 (C-9), 56.5 (C-5), 82.5 (C-15), 109.5 (C-17), 128.4 (C-2'), 136.9 (C-3'), 156.0 (C-16), 167.7 (C-1'), 177.7 (C-19).

12-Oxo-ent-kaur-9(11),16-dien-19-oic acid (**18**) [19]. Colorless crystals;^{1}H NMR ($CDCl_3$, 800 MHz) δ 1.04 (1H, *d*, *J*=10.7 Hz, CH_{2a}-3),1.17 (3H, *s*, CH_3-20), 1.26 (3H, *s*, CH_3-18), 1.28 (1H, *m*, CH_{2a}-1), 1.63 (1H, *ddd*, *J*=12.5, 9.4, 9.3 Hz, CH_{2a}-2), 1.63 (1H, *s*, CH-13), 1.69 (1H, *m*, CH_{2a}-7), 1.79 (1H, *d*, *J*=4.8Hz, CH_{2a}-15), 1.79 (1H, *ddd*, *J*=11.2, 4.8 Hz, CH_{2a}-6), 1.94 (1H, *dd*, *J*=11.0, 4.9 Hz, CH_{2b}-15), 1.95 (1H, *dd*, *J*=11.0, 4.9 Hz, CH_{2b}-1), 2.09 (1H, *dd*, *J*=9.8, 3.6 Hz, CH_{2b}-7), 2.14 (1H, *dd*, *J*=11.9, 2.1 Hz, CH_{2b}-6), 2.22 (1H, *d*, *J*=13.2 Hz, CH_{2b}-3), 2.40 (1H, *dt*, *J*=15.9, 2.9, 2.9 Hz,

CH_{2a}-14), 2.53 (1H, *d*, *J*=4.7 Hz, CH_{2b}-14), 3.39 (1H, *dd*, *J*=3.7, 1.4 Hz, CH-5), 5.00 (1H, *br s*, CH_{2a}-17), 5.25 (1H, *br s*, CH_{2b}-17), 5.76 (1H, *s*, CH-11); ^{13}C NMR ($CDCl_3$, 200 MHz) δ11.2 (C-17), 18.3 (C-6), 19.9 (C-2), 28.2 (C-18), 22.8 (C-20), 28.9 (C-7), 38.1 (C-3), 39.8 (C-1), 40.4 (C-4), 44.2 (C-14), 44.6 (C-8), 45.2 (C-10), 45.5 (C-13), 48.5 (C-15), 58.2 (C-5), 120.0 (C-11), 146.6 (C-16), 180.2 (C-9), 181.4 (C-19), 200.1 (C-12).

*Oleanolic acid (***19**) [20]: White Amorphous solid;^{1}H NMR ($CDCl_3$, 800 MHz) δ 0.80 (3H, *s*, CH_3-24), 0.81 (1H, *d*, *J*=7.2 CH-5), 0.82 (3H, *s*, CH_3-26), 0.94 (3H, *s*, CH_3-29), 0.96 (3H, *s*, CH_3-30), 0.96 (3H, *s*, CH_3-25), 0.98 (1H, *d*, *J*=6.6 Hz, CH_{2a}-1),1.01 (3H, *s*, CH_3-23), 1.10 (1H, *d*, *J*=4.7 Hz, CH_{2a}-15), 1.17 (1H, *dd*, *J*=3.6, 3.1 Hz, CH_{2a}-19), 1.19 (3H, *s*, CH_3-27), 1.22 (1H, *ddd*, 6.8, 6.1, 4.6 Hz, CH_{2a}-7), 1.32 (2H, *m*, CH_2-2/CH_{2a}-21), 1.44 (1H, *m*, CH_{2a}-6/CH_{2b}-7), 1.54 (1H, *m*, CH_{2b}-21), 1.57 (1H, *dd*, *J*=13.2, 5.1 Hz, CH_{2b}-6), 1.62 (2H, *m*, CH_{2b}-1/CH-9), 1.63 (2H, *m*, CH_{2a}-16/CH_{2a}-22), 1.76 (2H, *m*, CH_{2b}-15/CH_{2b}-19), 1.76 (1H, *ddd*, *J*=4.4, 4.4, 4.2 Hz, CH_{2b}-22), 1.90 (1H, *ddd*, *J*=4.2, 4.2, 2.6 Hz, CH_{2b}-11), 1.91 (1H, *dd*, *J*=8.9, 3.6 Hz, CH_{2a}-11), 2.05 (1H, *m*, CH_{2b}-16), 2.90 (1H, *dd*, *J*=14.0, 4.5 Hz, CH-18), 3.17 (1H, *dd*, *J*=11.3, 4.7 Hz, CH-3), 5.26 (1H, *t*, *J*=3.7, 3.7 Hz, CH-12); ^{13}C NMR ($CDCl_3$, 200 MHz) δ14.9 (C-25), 15.4 (C-24), 16.7 (C-26), 18.2 (C-6), 22.9 (C-30), 23.0 (C-16), 23.2 (C-11), 25.4 (C-27), 27.5 (C-23), 27.8 (C-15), 29.2 (C-2), 30.4 (C-20), 32.5 (C-22/ C-29), 32.8 (C-21), 33.6 (C-7),36.9 (C-10), 38.4 (C-8),38.6 (C-1), 39.3 (C-4), 41.4 (C-18), 41.6 (C-14),46.9 (C-17/C-19), 47.6 (C-9),55.9 (C-5), 77.1 (C-3), 122.1 (C-12), 140.0 (C-13), 177.9 (C-28).

3β-acetyloxy-olean-12-ene (**20**) [21,22]: White solid; ^{1}H NMR (CD_2Cl_2, 500 MHz) δ 0.88 (1H, *m*, CH_{2b}-22), 0.91 (6H, *s*, CH_3-25/CH_3-27), 0.92 (1H, *m*, CH-5), 0.93 (3H, *s*, CH_3-28), 1.01 (3H, *s*, CH_3-23), 1.02 (3H, *s*, CH_3-24), 1.16 (1H, *dd*, *J*=3.0, 2.8 Hz, CH_{2a}-19), 1.19 (6H, *s*, CH_3-29/CH_3-26), 1.26 (1H, *dt*, *J*=13.3, 3.3 Hz, CH_{2a}-1), 1.32 (2H, *dd*, *J*=9.2, 2.9 Hz, CH-2), 1.38 (3H, *s*, CH_3-30), 1.39 (1H, *td*, *J*=14.4, 14.0, 3.5 Hz, CH_{2a}-7), 1.39 (1H, *dd*, *J*=14.0, 3.5 Hz, CH_{2b}-21), 1.47 (2H, *m*, CH_{2b}-1/CH_{2a}-6), 1.59 (2H, *m*, CH_{2b}-6/CH_{2b}-7), 1.59 (2H, *m*, CH_{2a}-21/CH_{2a}-22), 1.65 (2H, *m*, CH-9/CH_{2a}-11), 1.65 (2H, *m*, CH_{2a}-16/CH_{2b}-19), 1.76 (2H, *m*, CH_2-15), 1.92 (2H, *m*, CH_{2b}-11/CH_{2b}-16), 1.99 (1H, *m*, CH-18), 2.06 (3H, *s*, CH_3-2'), 4.50 (1H, *dd*, *J*=6.2, 5.5 Hz, CH-3), 5.23 (1H, *t*, *J*=3.6 Hz, CH-12); ^{13}C NMR (CD_2Cl_2, 125 MHz) δ15.9 (C-24), 17.0 (C-23), 17.2 (C-25), 18.8 (C-6), 21.6 (C-2'), 24.0 (C-27), 24.2 (C-11/C-16), 26.3 (C-29), 26.7 (C-26), 27.5 (C-15), 28.3 (C-2), 28.7 (C-22), 33.0 (C-21), 33.1 (C-28),33.6 (C-7), 35.3 (C-20/C-30), 37.4 (C-10), 37.7 (C-8), 38.2 (C-1), 38.8 (C-18),40.4 (C-4), 42.3 (C-14), 47.3 (C-17), 47.8 (C-19), 48.1 (C-9), 55.8 (C-5), 81.3 (C-3), 122.3 (C-12), 146.7 (C-13), 171.2 (C-1');ESI-MS (30 eV), *m/z* (rel. int):469 $[M+H]^+$ (not observed), 391 (38), 371 (68), 329 (63), 185 (100), 175 (88), 182 (57), 149 (98), 205 (78), 251 (74), 259 (42).

Ent-kaur-9(11),16-diene (**21**) [23]. Colorless crystals; ^{1}H NMR (CD_2Cl_2, 600 MHz) δ 0.87 (1H, *m*, CH_{2a}-1), 0.99 (3H, *s*, CH_3-20), 1.07 (3H, *s*, CH_3-18), 1.08 (1H, *dd*, *J*=11.3, 5.1 Hz, CH_{2a}-3), 1.14 (1H, *dd*, *J*=4.1, 1.5 Hz, CH-5), 1.29 (3H, *s*, CH_3-19), 1.53 (1H, *ddd*, *J*=2.5, 1.8, 1.1 Hz, CH_{2a}-6), 1.54 (2H, *m*, CH_{2a}-12/CH-13),1.65 (1H, *dd*, *J*=1.2, 1.0 Hz, CH_{2a}-2), 1.74 (2H, *dd*, *J*=11.0, 8.5 Hz,

CH_2-7), 1.92 (1H, *ddd*, *J*=3.2, 2.1, 1.6 Hz, CH_{2b}-6), 2.01 (1H, *dd*, *J*=3.5, 1.5 Hz, CH_{2b}-1), 2.02 (1H, *dd*, *J*=1.5, 2.2 Hz, CH_{2b}-12), 2.08 (2H, *d*, *J*=2.7, CH_2-15), 2.23 (1H, *dt*, *J*=15.6, 2.6, 2.6 Hz, CH_{2a}-14), 2.49 (1H, *ddd*, *J*=1.7, 1.6, 1.6 Hz, CH_{2b}-2), 2.50 (1H, *dd*, *J*=11.4, 2.3 Hz, CH_{2b}-3), 2.67 (1H, *ddd*, *J*=5.2, 2.2, 2.1 Hz, CH_{2b}-14), 4.83 (1H, *d*, *J*=1.3 Hz, CH_{2a}-17), 4.96 (1H, *d*, *J*=1.4 Hz, CH_{2b}-17), 5.29 (1H, *br s*, CH-11); ^{13}C NMR (CD_2Cl_2, 150 MHz) δ 14.5 (C-20), 18.4 (C-2),20.2 (C-6),23.4 (C-18),28.0 (C-19),29.6 (C-12),37.9 (C-14),38.1 (C-3), 38.8 (C-10), 40.7 (C-1), 41.3 (C-13), 42.2 (C-4), 44.9 (C-8), 46.4 (C-7), 50.2 (C-15), 56.9 (C-5), 106.2 (C-17), 114.8 (C-11), 156.0 (C-16), 158.6 (C-9).

15 β-Hydroxy-ent-kaur-9(11),16-diene (**22**) [24]: Colorless crystals, ^{1}H NMR (Acetone-d_6, 600 MHz) δ1.09 (3H, *s*, CH_3-20), 1.05 (1H, *dd*, *J*=2.4, 1.6 Hz, CH_{2a}-3), 1.23 (3H, *s*, CH_3-18), 1.24 (2H, *d*, *J*=1.3 Hz, CH_2-1), 1.25 (1H, *dd*, *J*=3.1, 1.3 Hz, CH_{2b}-6), 1.51 (2H, *m*, CH_{2a}-2/CH_{2a}-14), 1.73 (1H, *m*, CH-5), 1.95 (2H, *m*, CH_{2b}-2/CH_{2a}-12), 2.08 (1H, *dd*, *J*=2.4, 1.8 Hz, CH_{2b}-14), 2.14 (1H, *m*, CH_{2b}-3), 2.31 (1H, *ddd*, 7.1, 4.0, 2.8 Hz, CH_{2b}-12), 2.63 (1H, *dd*, *J*=2.3, 3.7 Hz, CH_{2a}-6), 2.76 (1H, *ddd*, *J*=6.8, 5.1, 2.7 Hz, CH-13), 3.89 (1H, *br s*, CH-15), 4.78 (1H, *d*, *J*=1.8 Hz, , CH_{2a}-17), 4.91 (1H, *d*, *J*=2.3 Hz, CH_{2b}-17), 5.27 (1H, *m*, CH-11); ^{13}C NMR (Acetone-d_6, (150 MHz, ppm): 13.6 (C-20), 18.3 (C-2), 20.1 (C-6), 23.0 (C-18), 27.7 (C-14), 28.5 (C-19), 38.3 (C-3), 38.3 (C-12), 39.7 (C-10), 40.7 (C-1),43.1 (C-4), 45.7 (C-13), 46.8 (C-8),48.9 (C-7), 56.5 (C-5),72.2 (C-15),107.2 (C-17), 118.1 (C-11), 153.5 (C-9), 158.5 (C-16).

Methyl cinnamate (**23**) [25]. White solid;^{1}H NMR (Acetone-d_6, 500 MHz) δ7.78 (1H, *d*, *J*=8.6 HZ, CH-2), 6.88 (1H, *m*, CH-3), 6.96 (1H, *m*, CH-4), 7.78 (1H, *m*, CH-5), 6.96 (1H, *d*,*J*=8.6 Hz, CH-6), 6.32 (1H, *d*, *J*=15.6 Hz, CH-7), 7.56 (1H, *d*, *J*=15.6 Hz, CH-8), 3.73 (3H, *s*, MeO-9); ^{13}C NMR (Acetone-d_6, 125 MHz) δ 50.6 (MeO-19), 114.4 (C-6), 115.5 (C-2), 115.8 (C-5), 117.4 (C-3), 121.6 (C-4),126.6 (C-8),130.8 (C-1), 144.8 (C-7), 166.9 (C-9).

1.4. X-ray diffraction analyses

The single crystal X-ray data were collected using Agilent Super-Nova dual wavelength diffractometer with a micro-focus X-ray source and multilayer optics monochromatized Cu-Kα (λ = 1.54184 Å) radiation. Program *CrysAlisPro* [26] was used for the data collection and reduction. The intensities were corrected for absorption using analytical face index absorption correction method. The structures were solved with intrinsic phasing method (*SHELXT* [27]) and refined by full-matrix least squares on F^2 with *SHELXL-2018/3* [28]. Anisotropic displacement parameters were assigned to non-H atoms. All C-H hydrogen atoms were refined using riding models. Hydroxyl hydrogens were found from electron density maps and restrained to the proper distance from oxygen atom (0.84 Å). All hydrogen atoms were refined with U_{eq}(H) of 1.5×U_{eq}(C,O) for hydroxyl and terminal methyl groups or 1.2×U_{eq}(C) for other C-H groups. Further geometric least-squares restraints (s = 0.02) were applied to structures **4**, **6b** and **7** to obtain more chemically reasonable bond distances between disordered atoms. Anisotropic displacement parameters of few disordered or terminal atoms were restrained (s = 0.01, st = 0.02) to be more equal in structures **3**, **4**, **6b** and **7**.

Table S1. Crystal data and refinement parameters for **3**-**5** and **7**.

	**3**	**4**	**5**	**7**[a]
CCDC number	1868318	1868319	1868321	1868323
Formula	$C_{25}H_{36}O_5$	$C_{26}H_{38}O_5$	$C_{25}H_{36}O_5$	$C_{25}H_{36}O_4$
Formula weight	416.54	430.56	416.54	400.54
T [K]	120(2)	120(2)	170(2)	120(2)
Crystal system	orthorhombic	orthorhombic	hexagonal	monoclinic
Space group (number)	$P2_12_12_1$(19)	$P2_12_12_1$(19)	$P6_522$ (179)	$C2$ (5)
Unit cell dimensions				
a [Å]	8.68202(13)	8.31056(11)	14.07860(10)	11.1698(3)
b [Å]	10.84059(15)	10.89983(12)	14.07860(10)	8.5815(3)
c [Å]	23.5621(3)	25.1640(3)	39.5776(4)	23.5527(8)
β [°]	90	90	90	92.080(3)
V [Å^3]	2217.62(5)	2279.45(5)	6793.58(12)	2256.12(13)
Z	4	4	12	4
ϱ_{calc}	1.248	1.255	1.222	1.179
μ [mm^{-1}]	0.684	0.681	0.670	0.618
F(000)	904	936	2712	872
Crystal size [mm]	0.18×0.14×0.13	0.28×0.23×0.09	0.24×0.10×0.09	0.17×0.14×0.03
θrange[°]	3.752 - 74.175	3.513- 74.341	3.794- 74.465	3.756- 73.896
Refl. collected	15950	39010	25659	7630
Independent refl.	4461	4596	4625	4415
R_{int}	0.0281	0.0534	0.0255	0.0243
Reflections[I>2σ(I)]	4307	4433	4411	3987
Completeness to θ	100.0	100.0	100.0	99.8
Max. /min. transm.	0.952 / 0.933	0.956/ 0.895	0.953/ 0.903	0.984/ 0.937
Restraints / parameters	14 / 280	175 / 343	0 / 279	58 / 281
Goodness-of-fit on F^2	1.027	1.067	1.029	1.047
Final Rindices [I>2σ(I)]	R1 = 0.0327, wR2 = 0.0856	R1 = 0.0417, wR2 = 0.1153	R1 = 0.0323, wR2 = 0.0863	R1 = 0.0464, wR2 = 0.1224
Rindices (all data)	R1 = 0.0342, wR2 = 0.0872	R1 = 0.0429, wR2 = 0.1167	R1 = 0.0343, wR2 = 0.0881	R1 = 0.0520, wR2 = 0.1285
Flack parameter	0.07(6)	-0.08(7)	0.01(6)	0.1(3)[a]
Largest peak/hole [e.Å^{-3}]	0.376 / -0.181	0.499/ -0.244	0.147/ -0.134	0.282 / -0.199

[a] Refined as a 2-component inversion twin: BASF = 0.14. Standard uncertainty of Flack parameter too high and reliable absolute structure determination was not possible.

Table S2. Crystal data and refinement parameters for **6**, **6b** and **8**.

	**6**	**6b**	**8**
CCDC number	1868320	1868324	1868322
Formula	$C_{20}H_{28}O_2$	$C_{20}H_{30}O_2$	$C_{20}H_{32}O_2$
Formula weight	300.42	302.44	304.45
T [K]	120(2)	120(2)	120(2)
Crystal system	monoclinic	orthorhombic	orthorhombic
Space group (number)	$P2_1$ (4)	$P2_12_12_1$ (19)	$P2_12_12_1$ (19)
Unit cell dimensions			
a [Å]	10.67468(12)	17.1810(2)	11.16620(10)
b [Å]	11.35748(12)	17.3156(2)	14.0602(2)
c [Å]	14.10077(16)	23.4406(3)	22.4854(3)
β [°]	99.0578(10)	90	90
V [Å^3]	1688.22(3)	6973.56(15)	3530.19(8)
Z	4 (Z' = 2)	16 (Z' = 4)	8 (Z' = 2)
ϱ_{calc}	1.182	1.152	1.146
μ [mm^{-1}]	0.574	0.557	0.550
F(000)	656	2656	1344
Crystal size [mm]	0.30×0.21×0.16	0.42×0.16×0.12	0.23×0.14×0.06
θrange[°]	4.194 - 74.268	3.624 - 76.805	3.708- 74.202
Refl. collected	11890	140940	26113
Independentrefl.	6618	14476	7096
R_{int}	0.0244	0.0607	0.0259
Reflections[I>2σ(I)]	6366	13376	6706
Completeness to θ	99.8	99.9	100.0
Max. /min. transm.	0.925/ 0.895	0.950/ 0.854	0.965/ 0.909
Restraints / parameters	3 / 403	215 / 867	2 / 403
Goodness-of-fit on F^2	1.035	1.031	1.025
Final Rindices [I>2σ(I)]	R1 = 0.0340, wR2 = 0.0849	R1 = 0.0517, wR2 = 0.1335	R1 = 0.0383, wR2 = 0.1021
Rindices (all data)	R1 = 0.0355, wR2 = 0.0872	R1 = 0.0555, wR2 = 0.1370	R1 = 0.0408, wR2 = 0.1044
Flack parameter	-0.06(9)	0.10(5)	-0.05(7)
Largest peak/hole [e.Å^{-3}]	0.225/ -0.158	0.337/ -0.329	0.209/ -0.190

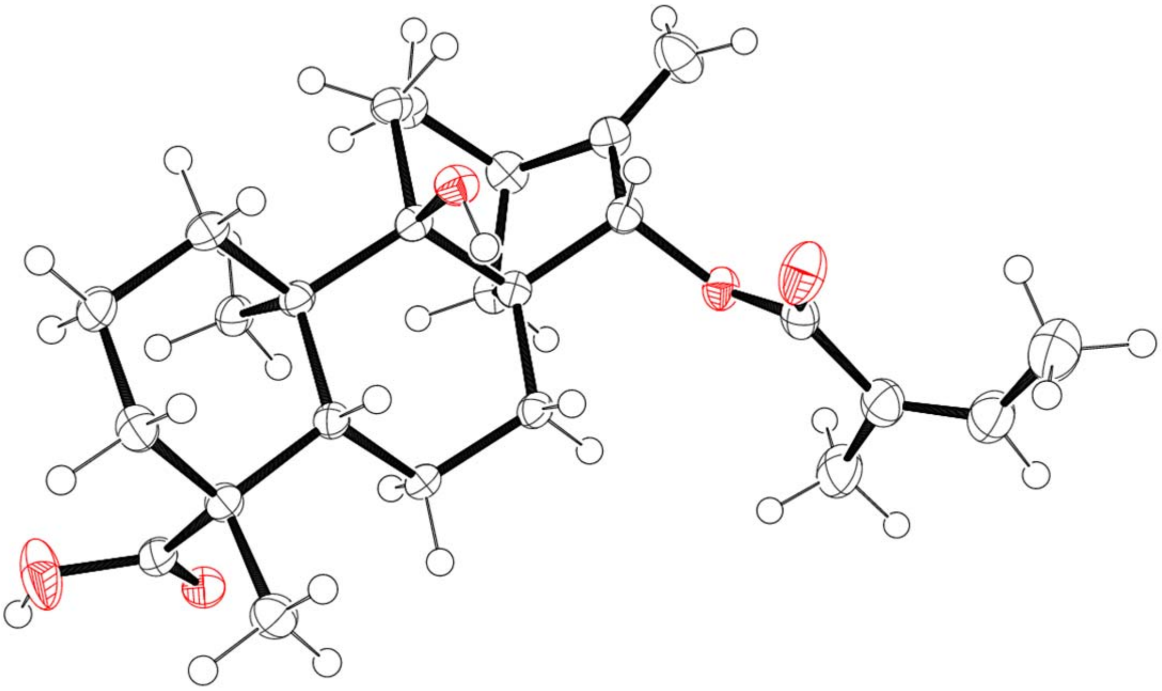

Figure S15. Thermal ellipsoid diagram of **3** (ellipsoid probability 50 %).

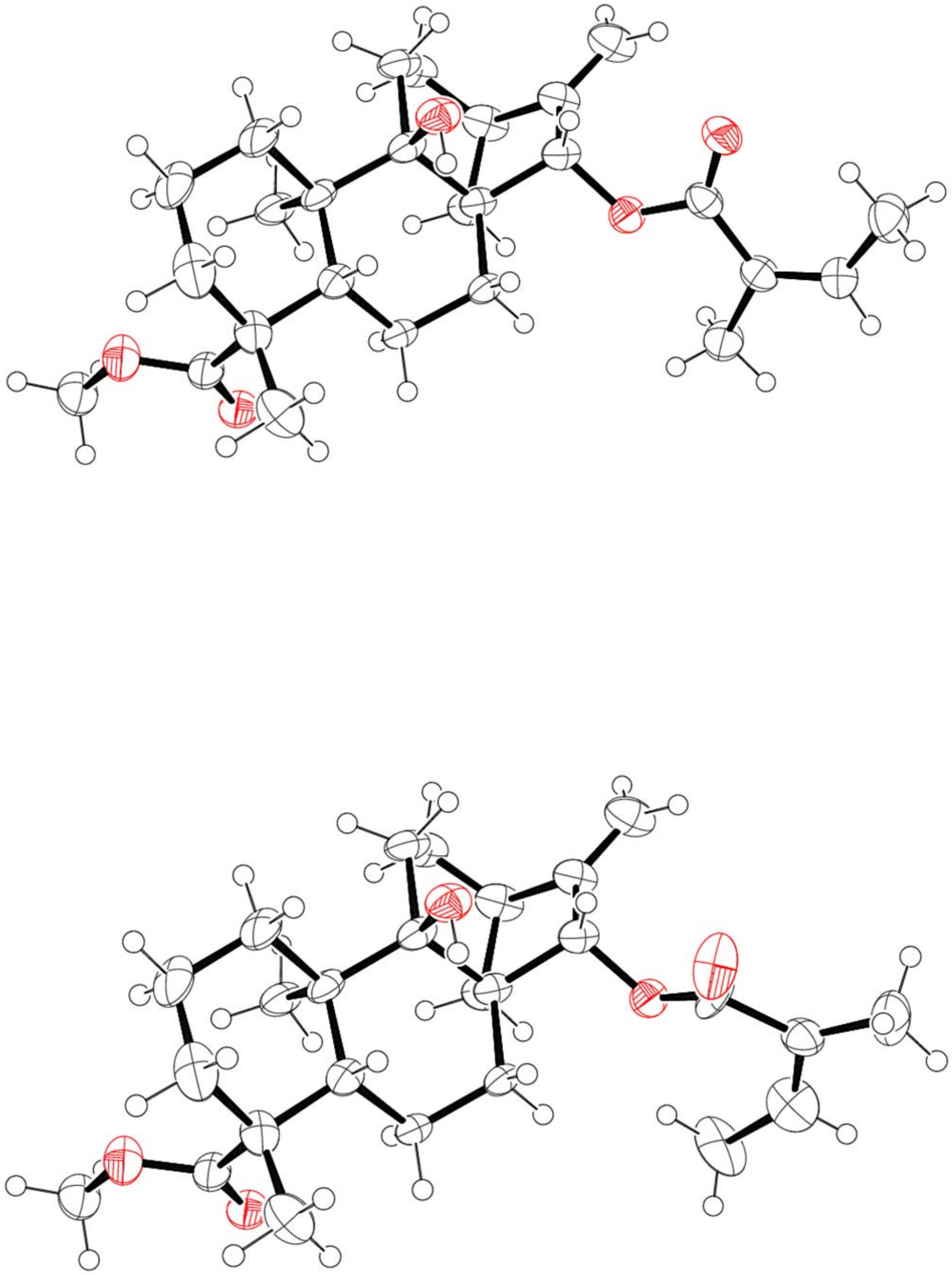

Figure S16. Thermal ellipsoid diagrams of **4** (ellipsoid probability 50 %) showing two different and equal (~1:1) spatial orientations of (*Z*)-2-methylbut-2-enoyl group.

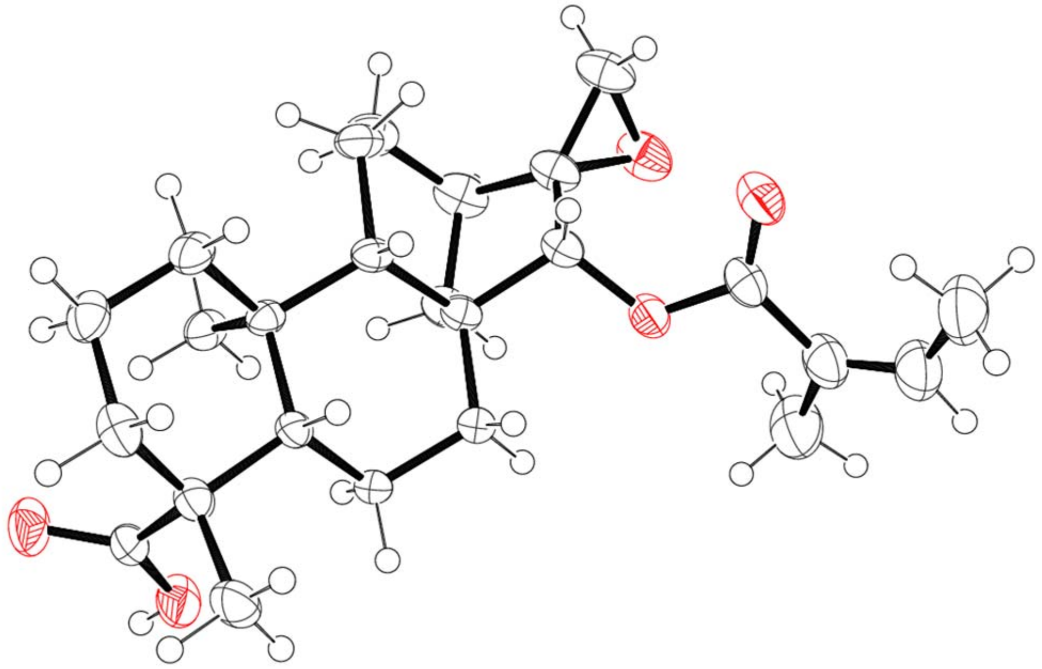

Figure S17. Thermal ellipsoid diagram of **5** (ellipsoid probability 50%).

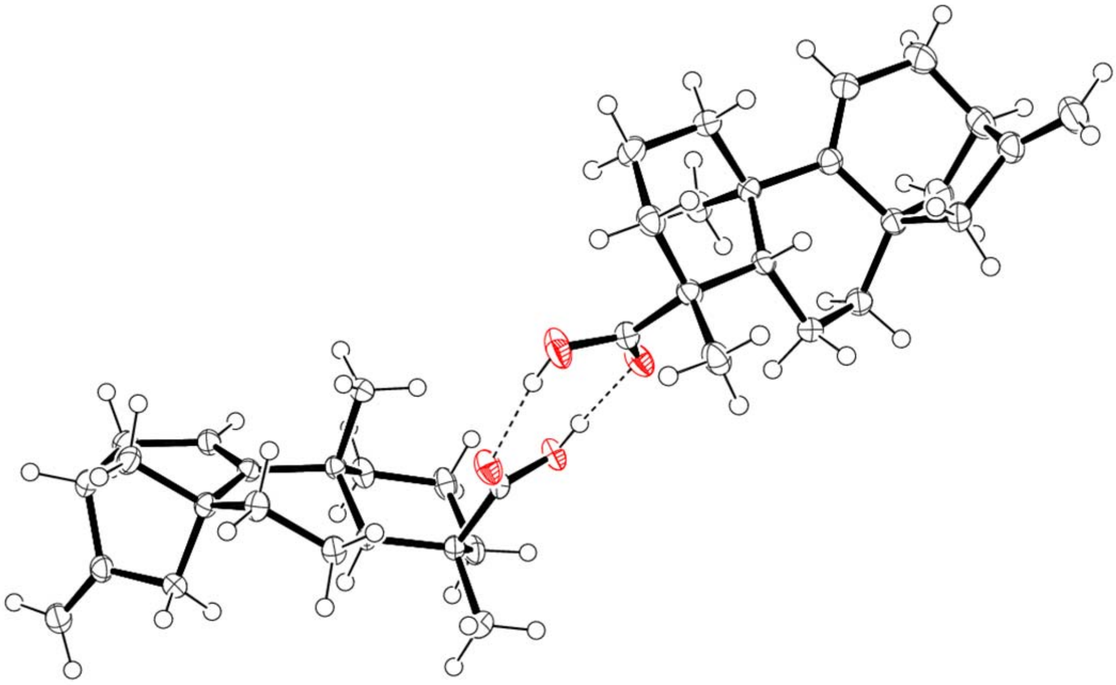

Figure S18. Thermal ellipsoid diagram of **6** (ellipsoid probability 50 %) showing both crystallographically independent molecules in asymmetric unit (AU) and hydrogen bonding (dashed) between them. This corresponds the reported structure by Reynolds. *et. al.* [21].

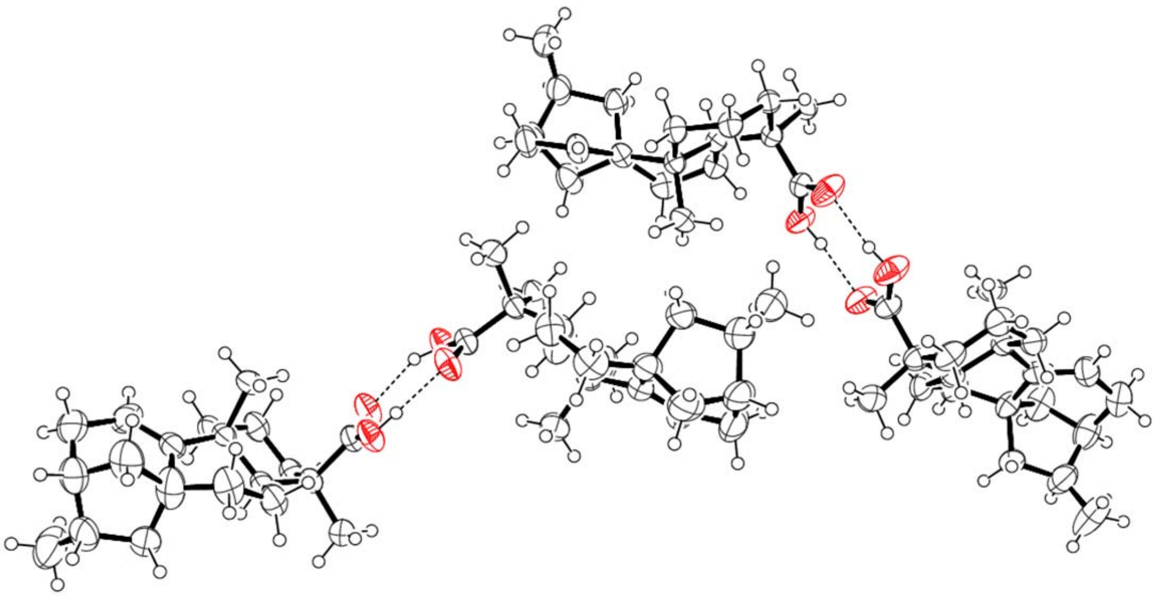

Figure S19. Thermal ellipsoid diagram of **6b** (ellipsoid probability 50 %) showing twohydrogen-bonded molecular pairs composed by four crystallographically independent molecules in AU.

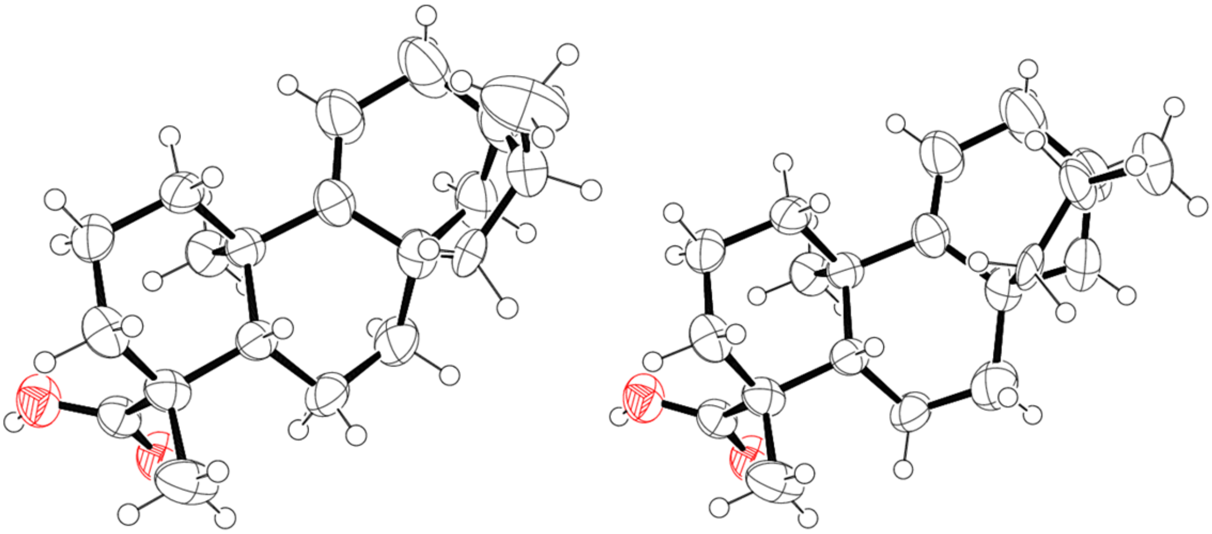

Figure S20. Two different conformations (~90:10 left/right) in aliphatic rings of **6b**.

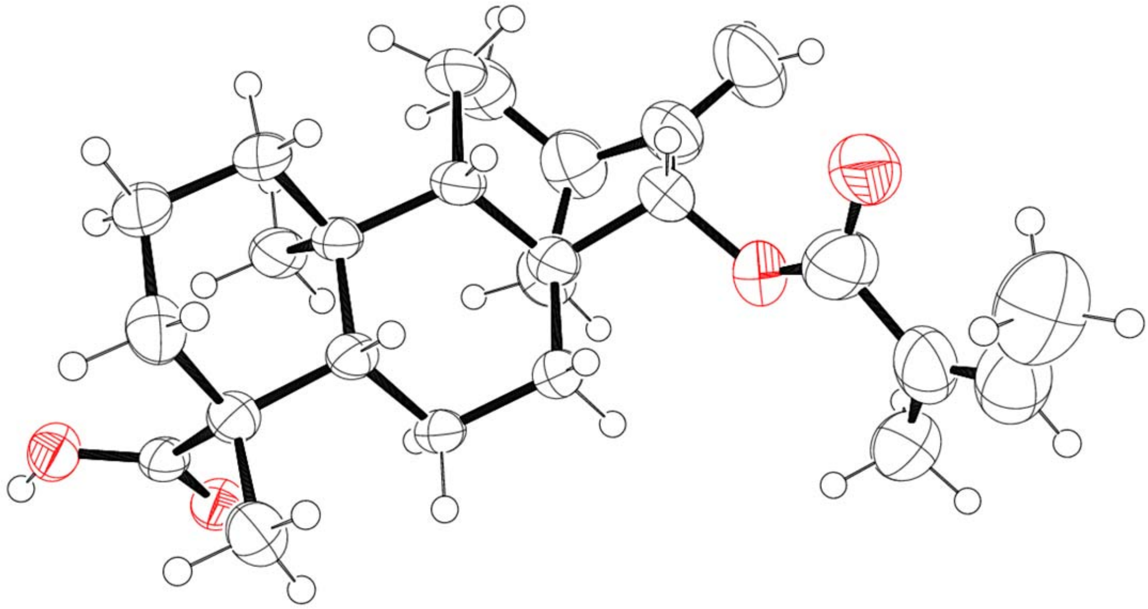

Figure S21. Thermal ellipsoid diagram of **7** (ellipsoid probability 50 %).

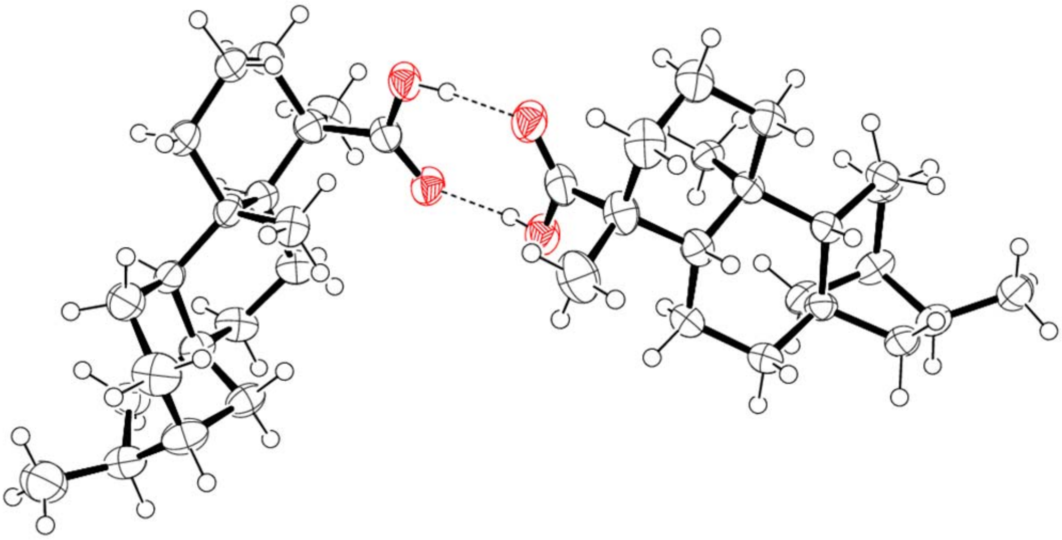

Figure S22. Thermal ellipsoid diagram of **8** (ellipsoid probability 50 %) showing ahydrogen-bonded molecular pair composed by two crystallographically independent molecules in AU.

Table S3. Hydrogen bonding geometries in structures **3-8**.

D-H...A	*d*(D-H) [Å]	*d*(H...A) [Å]	*d*(D...A) [Å]	<(DHA) [°]
**3**				
O(19b)-H…O(21)[a]	0.83(2)	1.92(3)	2.690(2)	155(4)
O(9)-H…O(19a)[b]	0.85(2)	2.05(2)	2.835(2)	152(3)
**4**				
O(9)-H…O(19)[a]	0.90(2)	2.03(3)	2.839(3)	149(3)
**5**				
O(19b)-H...O(19a)[c]	0.82(6)	1.78(6)	2.594(3)	171(6)
O(19a)-H...O(19b)[c]	0.90(5)	1.74(5)	2.634(3)	176(6)
**6**				
O(19b)-H...O(19c)[d]	0.85(2)	1.78(2)	2.625(2)	177(3)
O(19d)-H...O(19a)[d]	0.83(2)	1.81(2)	2.637(2)	171(4)
**6b**				
O(19b)-H...O(O19c)[d]	0.84(3)	1.75(3)	2.586(3)	176(11)
O(19a)-H...O(19d)[d]	0.85(3)	1.82(4)	2.658(3)	167(10)
O(19d)-H...O(19a)[d]	0.84(3)	1.84(4)	2.658(3)	163(10)
O(19c)-H...O(19b)[d]	0.85(3)	1.78(5)	2.586(3)	156(10)
O(19f)-H...O(19g)[e]	0.86(2)	1.82(3)	2.662(3)	168(5)
O(19h)-H...O(19e)[e]	0.88(3)	1.79(3)	2.633(3)	162(5)
**7**				
O(19b)-H...O(19a)[f]	0.81(3)	1.83(3)	2.639(2)	176(10)
O(19a)-H...O(19b)[f]	0.82(3)	1.82(3)	2.639(2)	171(9)
**8**				
O(19b)-H...O(19c)[g]	0.84(2)	1.79(2)	2.617(2)	170(3)
O(19d)-H...O(19a)[g]	0.84(2)	1.80(2)	2.632(3)	167(4)

[a] -x+1,y+1/2,-z+3/2, [b] -x+1,y-1/2,-z+3/2, [c] -y+2,-x+2,-z+7/6 (half occupancy for H-atom positions), [d] O(19c) and O(19d) atoms of second molecule in AU (half occupancy for H-atom positions), [e] O(19e) and O(19f) are atoms of third as well as O(19g) and O(19h) are atoms of fourth molecule in (AU), [f] -x+1,y,-z+1 (half occupancy for H-atom positions), [g] O(19c) and O(19d) atoms of second molecule in AU

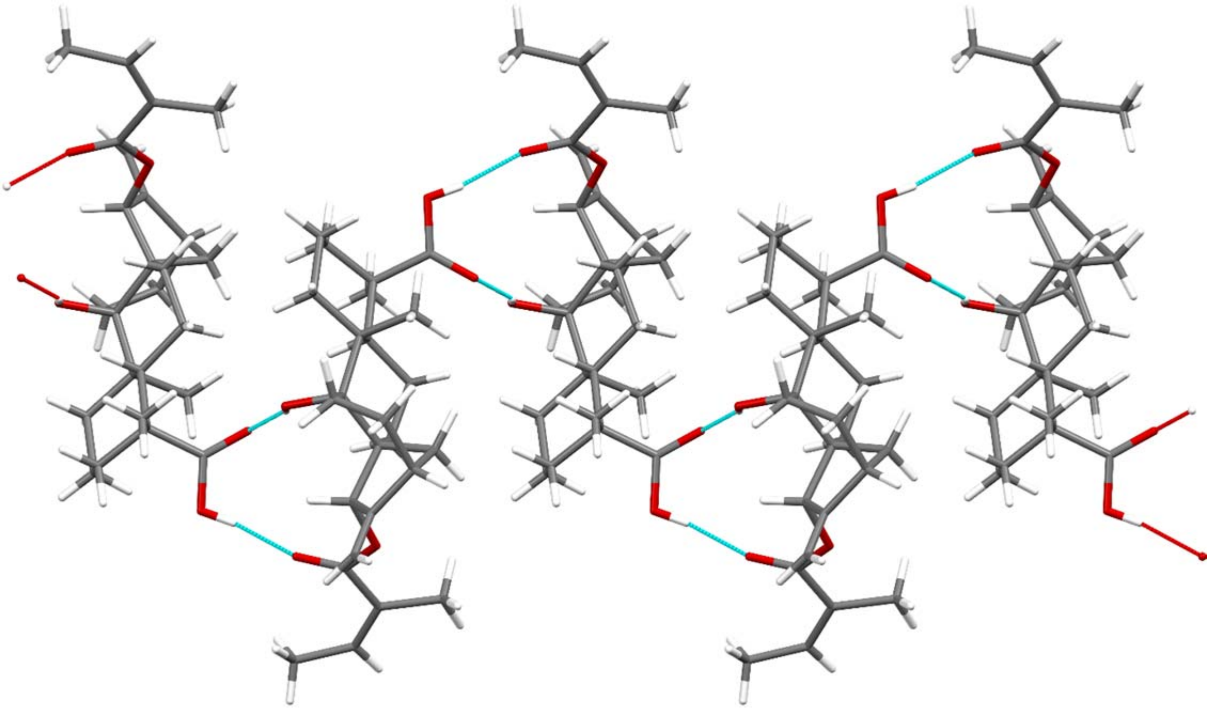

Figure S23. A motif along crystallographic *b* axis formed by O(9)-H···O(19a) and O(19b)-H···O(21) intermolecular hydrogen bonds (viewed along *a*) in **3**. Hydrogen bonding contacts are shown as turquoise (expanded) or red (hanging) stick model.

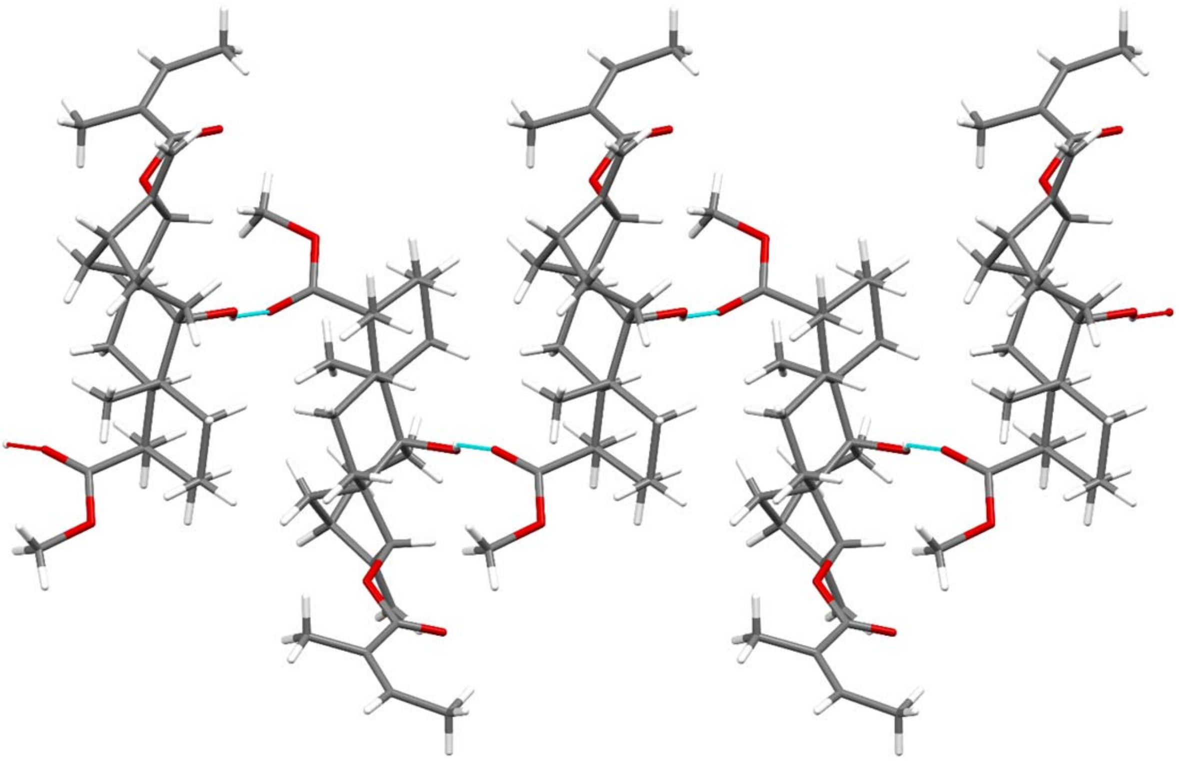

Figure S24. A motif along crystallographic *b* axis formed by O(9)-H···O(19a) intermolecular hydrogen bonds (viewed along *a*) in **4**. Hydrogen bonding contacts are shown as turquoise (expanded) or red (hanging) stick model.

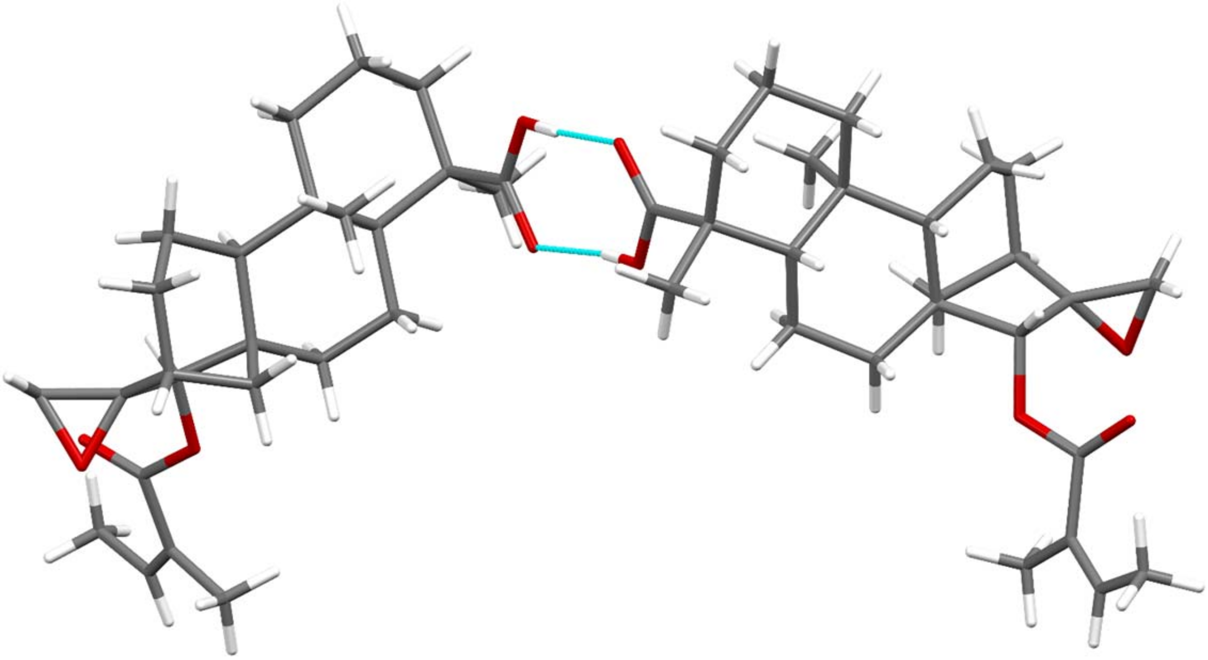

Figure S25. Ahydrogen-bonded molecular pair composed by two molecules of **5**.Hydrogen bonding contacts are shown as turquoise stick model.

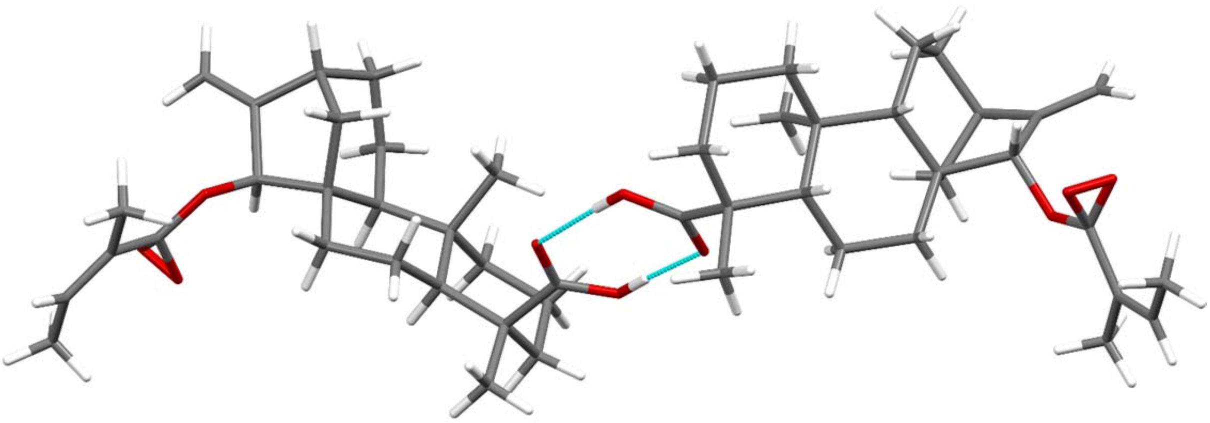

Figure S26. Ahydrogen-bonded molecular pair composed by two molecules of **7**.Hydrogen bonding contacts are shown as turquoise stick model.

1.5. References

1. Duan, H.; Takaishi, Y.; Momota, H.; Ohmoto, Y.; Taki T., Jia, Y.; Li, D. Immunodepressive diterpenoids from *Tripterygium wilfordii*. *J. Nat. Prod*. **1999**, *62*, 1522-1525.
2. Lee, I.S.; Jin, W.Y.; Zhang, X.; Hung, T.M.; Song, K.S.; Seong, Y.H.; Bae, K. Cytotoxic and COX-2 inhibitory constituents from the aerial parts of *Aralia cordata*. *Arch Pharm. Res.* **2006**, *29*, 548-555.
3. Müller, S.; Tirapelli, C.R.; de Oliveira, A.M.; Murillo, R.; Castro, V.; Merfort, I. Studies of ent-kaurane diterpenes from *Oyedaea verbesinoides* for their inhibitory activity on vascular smooth muscle contraction. *Phytochemistry* **2003**, *63*, 391-396.
4. Delgado, G.; Vivar, A.R.D. *Ent*-kaurenoid methyl esters from *Viguiera stenoloba*, structural revision of stenolobin and its biomimetic conversion to zoapatlin. *Chem. Lett.* **1984**, *13*, 1237-1240.
5. Wafo, P.; Kamdem, R.S.; Ali, Z.; Anjum, S.; Begum, A.; Oluyemisi, O.O.; Khan, S.N.; Ngadjui, B.T.; Etoa, X.F.; Choudhary, M.I. Kaurane-type diterpenoids from *Chromoleana odorata*, their X-ray diffraction studies and potent α-glucosidase inhibition of 16-kauren-19-oic acid. *Fitoterapia* **2011**, *82*, 642-646.
6. Arciniegas, A.; Pérez-Castorena, A.L.; Meléndez-Aguirre, M.; Ávila, J.G.; García-Bores, A.M.; Villaseñor, J.L.; Romod.V.A. Chemical composition and antimicrobial activity of *Ageratina deltoidea*. *Chem. Biodivers.* **2018**, *15*, e1700529.
7. Boeck, P.; Sá, M.M.; Souza, B.S.D.; Cercená, R.; Escalante, A.M.; Zachino, S.A.; Cechinel F.V.; Yunes, R.A. A simple synthesis of kaurenoic esters and other derivatives and evaluation of their antifungal activity. *J. Brazil. Chem. Soc.* **2005**, *16*, 1360-1366
8. El Marsni, Z.; Torres, A.; Varela, R.M.; Molinillo, J.M.; Casas, L.; Mantell, C.; Martinez, E.J.D.L.O.; Macias, F.A. Isolation of bioactive compounds from sunflower leaves (*Helianthus annuus* L.) extracted with supercritical carbon dioxide. *J. Agric. Food Chem.* **2015**, *63*, 6410-6421.
9. Tincusi, B.M.; Jiménez, I.A.; Bazzocchi, I.L.; Moujir, L.M.; Mamani, Z.A.; Barroso, J.P.; Ravelo, A.G.; Hernandez, B.V. Antimicrobial terpenoids from the oleoresin of the peruvian medicinal plant *Copaifera paupera*. *Planta Med.* **2002**, *68*, 808-812.
10. Pinto, A.C.; Prado, S.K.D.; Pinchin, R. Two kaurenes from *Vellozia caput-ardeae*. *Phytochemistry* **1981**, 20, 520-521.
11. Aráoz, M.V.C.; Mercado, M.I.; Grau, A.; Catalán, C.A. *Ent*-kaurane derivatives from the root cortex of Yacon and other three *Smallanthus* species (Heliantheae, Asteraceae). *Biochem Syst. Ecol.* **2010**, *38*, 1042-1048.
12. Chen, Q.; Lin, H.; Wu, X.; Song, H.; Zhu, X. Preparative separation of six terpenoids from *Wedelia prostrata* Hemsl. By two-step high-speed counter-current chromatography. *J. Liq. Chromatogr. Relat. Technol.* **2018**, *41*, 408-414.
13. Peña, A.; Alarcón, L.; Baptista, J.G.; Aparicio, R.; Villasmil, T.; Usubillaga, A. A phytochemical analysis of *Espeletia nana* Cuatrec. A midget Espeletiinae from Paramo Ortiz, Venezuela. *Av. Quim.* **2012**, *7*, 187-192.
14. Dias, J.R.; Gao, H. ^{13}C Nuclear Magnetic Resonance data of lanosterol derivatives—profiling the steric topology of the steroid skeleton via substituent effects on its ^{13}C NMR. *Spectrochim. Acta Part A: Mol. Biomol. Spectrosc.* **2009**, *74*, 1064-1071.
15. Chaturvedula, V.S.P.; Prakash, I. Isolation of stigmasterol and β-sitosterol from the dichloromethane extract of *Rubus suavissimus*. *Int. Curr. Pharm. J.* **2012**, *1*, 239-242.
16. Zhou, Y.; Yang, B.; Liu, Z.; Jiang, Y.; Liu, Y.; Fu, L.; Wang, X.; Kuang, H. Cytotoxicity of triterpenes from green walnut husks of *Juglans mandshurica* maxim. in HepG-2 cancer cells. *Molecules* **2015**, *20*, 19252-19262.
17. Reisch, J.; Hussain, R.A.; Krebs, B.; Dartmann, M. Natural product chemistry, part 100: The structure of carissone, $C_{15}H_{24}O_2$. *Monatsh. Chem.* **1990**, *121*, 941-944.
18. Ohno, N.; Mabry, T.J.; Zabelt, V.; Watson, W.H. Tetrachyrin, a new rearranged kaurenoid lactone, and diterpene acids from *Tetrachyron orizabaensis* and *Helianthus debilis*. *Phytochemistry*, **1979**, *18*, 1687-1689.
19. Huang, W.; Liang, Y.; Wang, J.; Li, G.; Wang, G.; Li, Y.; Chung, H.Y. Anti-angiogenic activity and mechanism of kaurane diterpenoids from *Wedelia chinensis*. *Phytomedicine* **2016**, *23*, 283-292.
20. Seebacher, W.; Simic, N.; Weis, R.; Saf, R.; Kunert, O. Complete assignments of ^{1}H and ^{13}C NMR resonances of oleanolic acid, 18α-oleanolic acid, ursolic acid and their 11-oxo derivatives. *Magn. Reson. Chem.* **2003**, *41*, 636-638.
21. Faleye, F.J. Terpenoid constituents of *Aspilia africana* [Pers.] C.D. Adams leaves. *Int. J. Pharm. Sci. Rev. Res.* **2012**, *13*, 138-142.

22. Ogihara, K.; Iraha, R.; Higa, M.; Yogi, S. Studies on constituents from the twigs of *Messerschmidia argentea* II. *Bulletin of the College of Science, University of the Ryukyus* **1997**, 64, 53-59.
23. Zhang, H.; Wynne, G.; Mander, L.N. Synthesis of *ent*-9α, 15α-cyclokaurene from grandiflorenic acid. *ARKIVOC* **2001**, *8*, 40-58.
24. Nagashima, F.; Kondoh, M.; Fujii, M.; Takaoka, S.; Watanabe, Y.; Asakawa, Y. Novel cytotoxic kaurane-type diterpenoids from the New Zealand liverwort *Jungermannia* species. *Tetrahedron* **2005**, *61*, 4531-4544.
25. Gao, L.; Xu, X.; Nan, H.; Yang, J.; Sun, G.; Wu, H.; Zhong, M. Isolation of cinnamic acid derivatives from the root of *Rheum tanguticum* Maxim. Ex balf. And its significance. *J. Med. Plants Res.* **2012**, *6*, 929-931.
26. *CrysAlisPro* (Version 1.171.38.41.), Rigaku Oxford Diffraction, 2015.
27. Sheldrick, G.M. *ActaCrystallogr., Sect. A* **2015**, *71*, 3-8.
28. Sheldrick, G.M. *ActaCrystallogr., Sect. C* **2015**, *71*, 3-8.
